# Supplementary figures and images for: Extracellular matrix-driven metabolic control of pancreatic endocrine lineage allocation
Source: EMBO Rep. 2025 Oct 27;26(23):5877–904. doi: 10.1038/s44319-025-00610-6 (PMC12678434; doi:10.1038/s44319-025-00610-6)

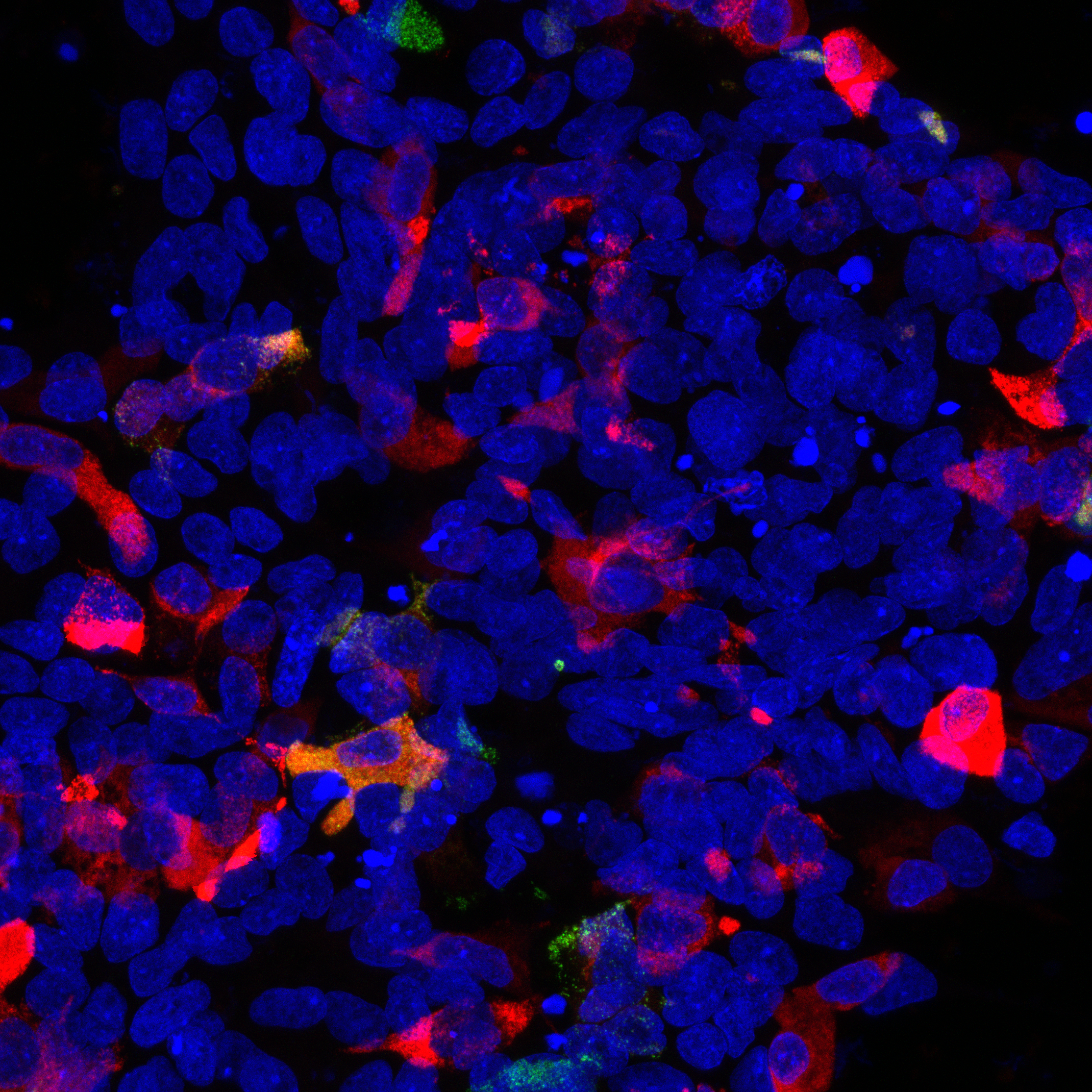

Supplement: Supplementary file 2 — Source data Fig. 1 [file 44319_2025_610_MOESM2_ESM.zip › Figure 1/1A/Fig1A_MIP_FN.png]

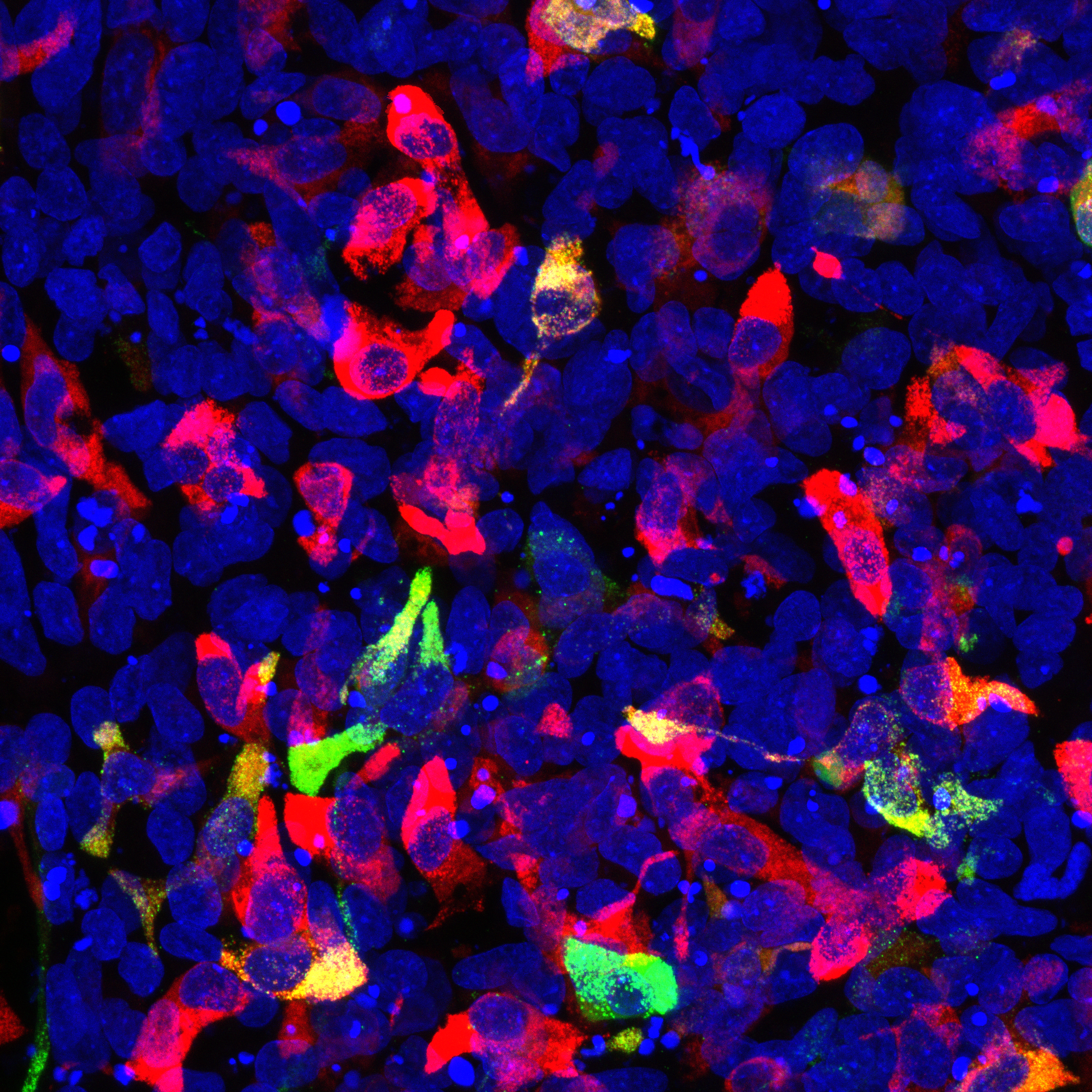

Supplement: Supplementary file 2 — Source data Fig. 1 [file 44319_2025_610_MOESM2_ESM.zip › Figure 1/1A/Fig1A_MIP_LN111.png]

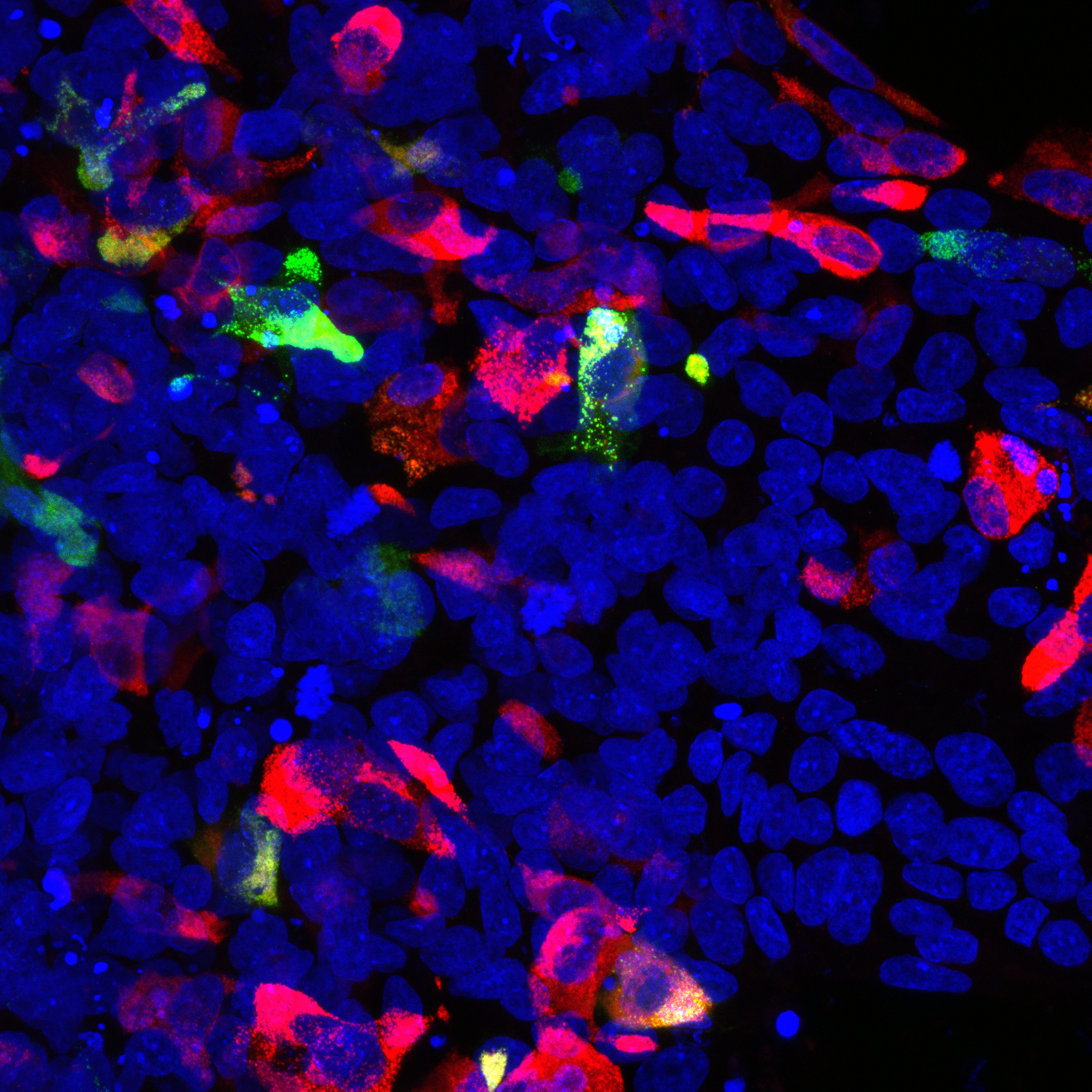

Supplement: Supplementary file 2 — Source data Fig. 1 [file 44319_2025_610_MOESM2_ESM.zip › Figure 1/1A/Fig1A_MIP_LN121.png]

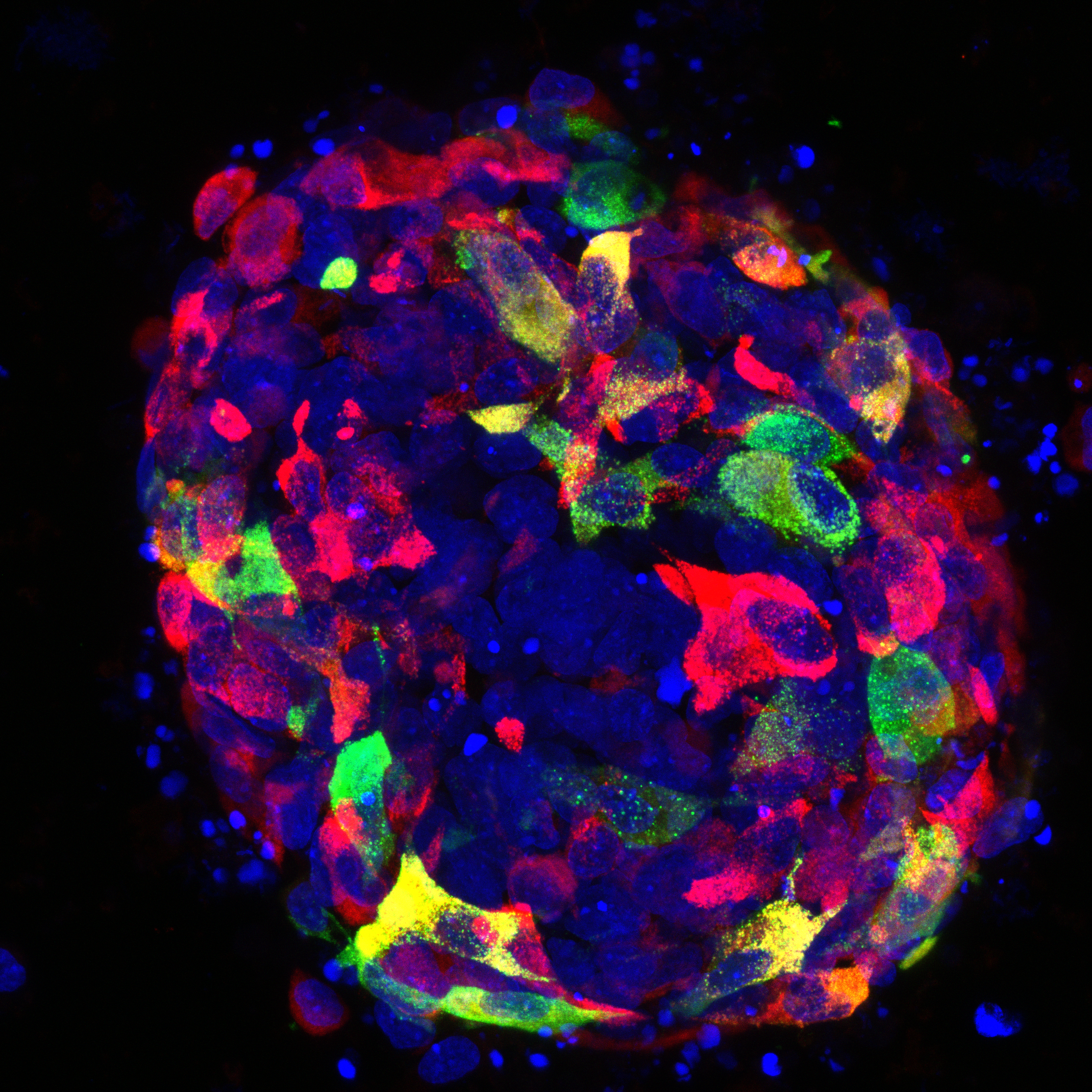

Supplement: Supplementary file 2 — Source data Fig. 1 [file 44319_2025_610_MOESM2_ESM.zip › Figure 1/1A/Fig1A_MIP_LN211.png]

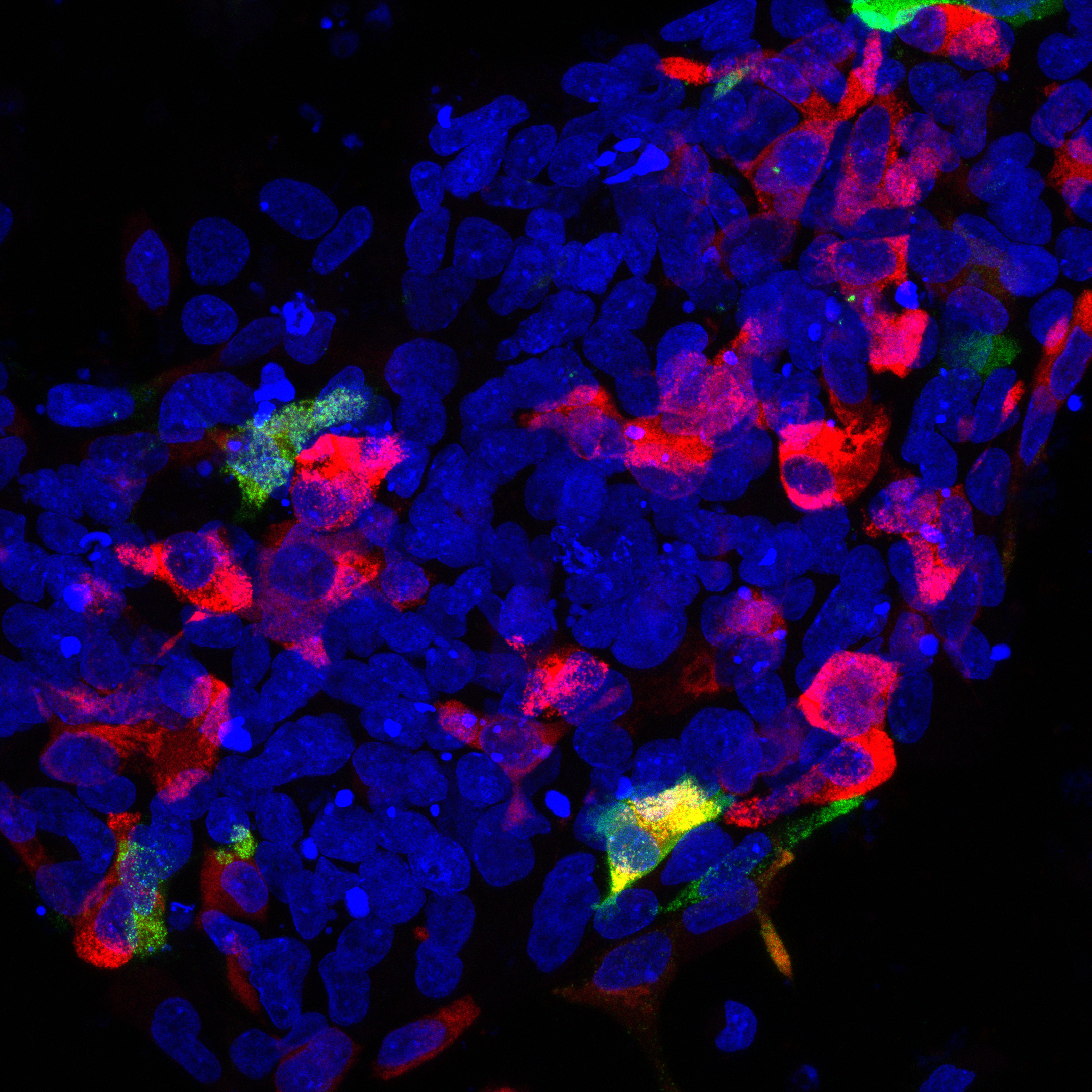

Supplement: Supplementary file 2 — Source data Fig. 1 [file 44319_2025_610_MOESM2_ESM.zip › Figure 1/1A/Fig1A_MIP_LN221.png]

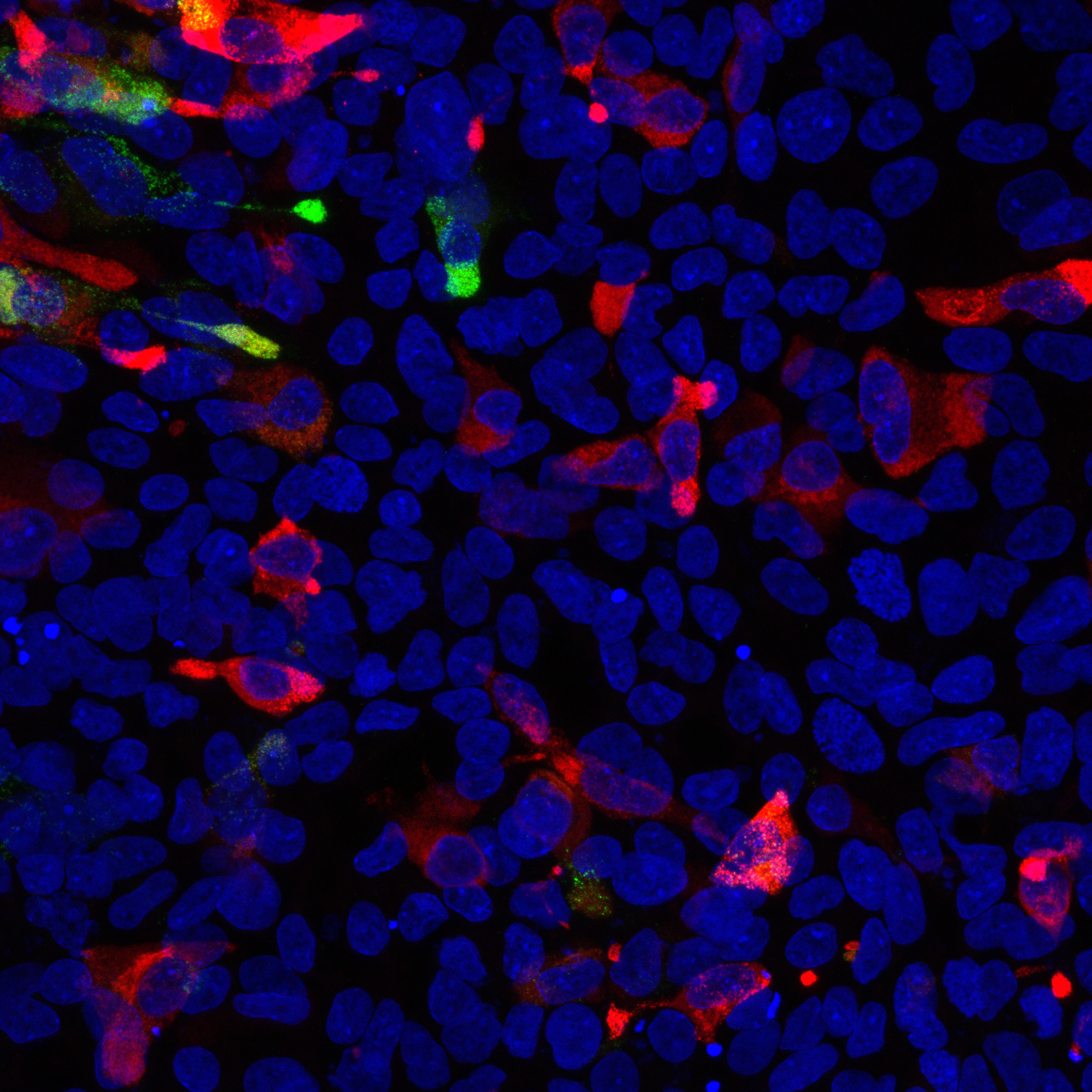

Supplement: Supplementary file 2 — Source data Fig. 1 [file 44319_2025_610_MOESM2_ESM.zip › Figure 1/1A/Fig1A_MIP_LN332.png]

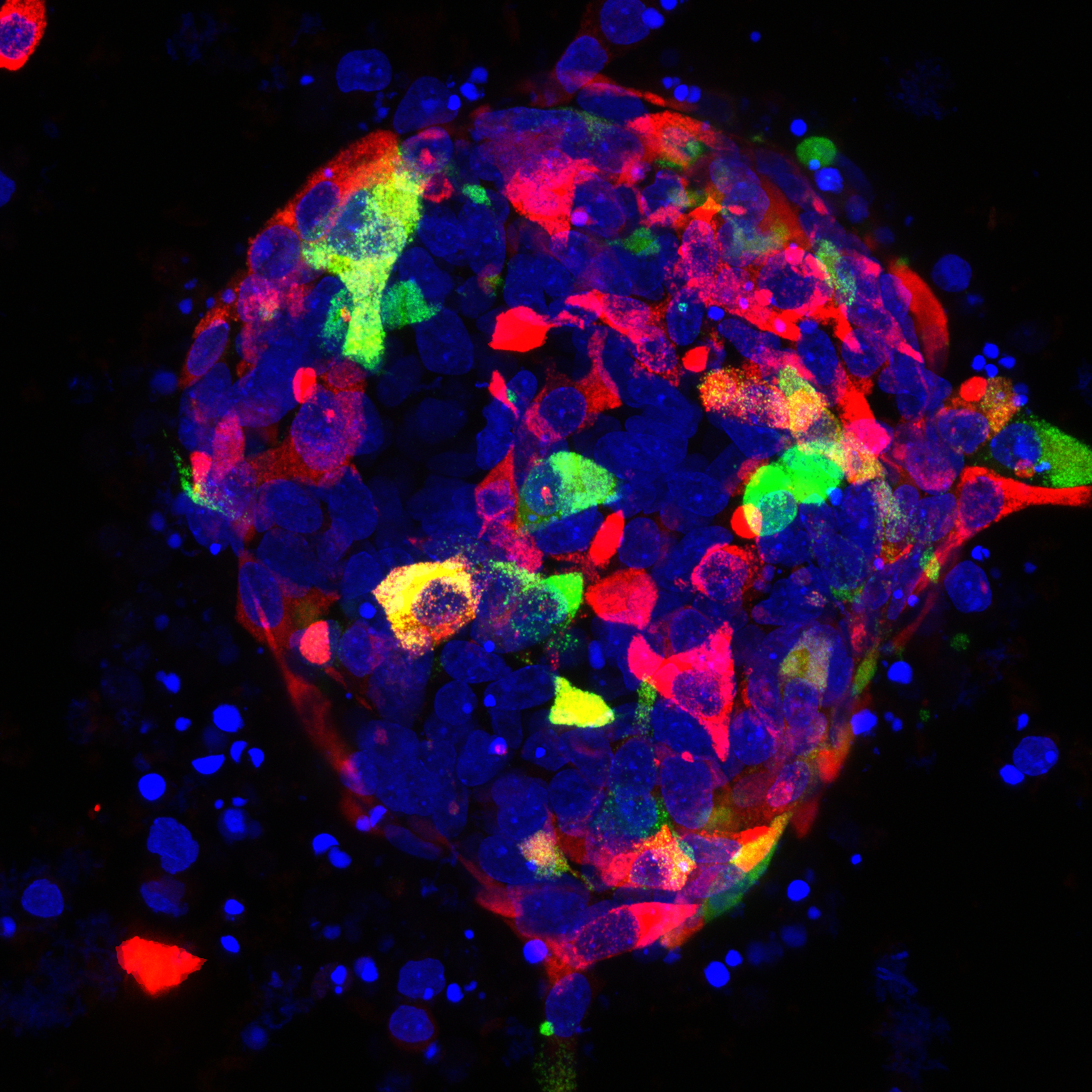

Supplement: Supplementary file 2 — Source data Fig. 1 [file 44319_2025_610_MOESM2_ESM.zip › Figure 1/1A/Fig1A_MIP_LN411.png]

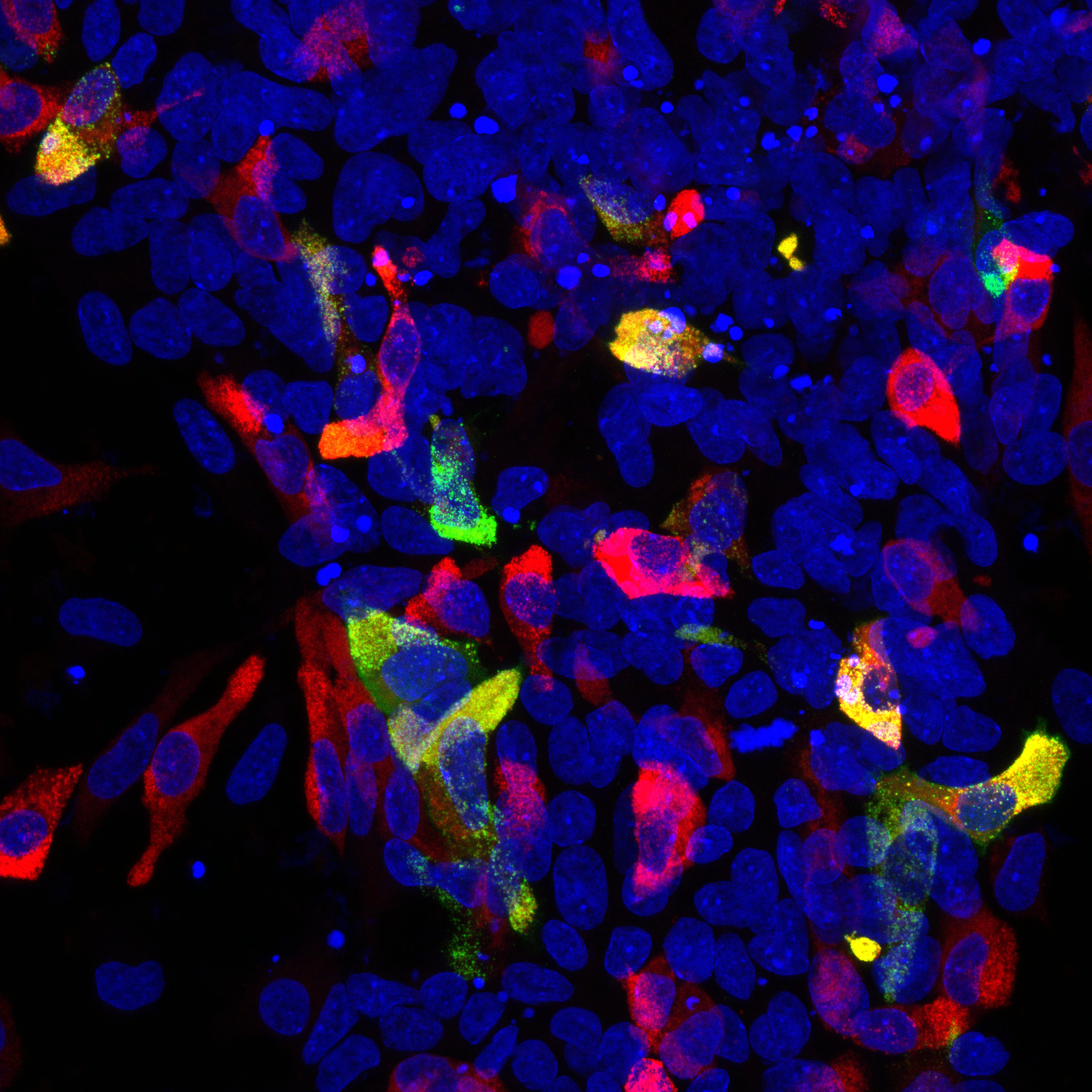

Supplement: Supplementary file 2 — Source data Fig. 1 [file 44319_2025_610_MOESM2_ESM.zip › Figure 1/1A/Fig1A_MIP_LN421.png]

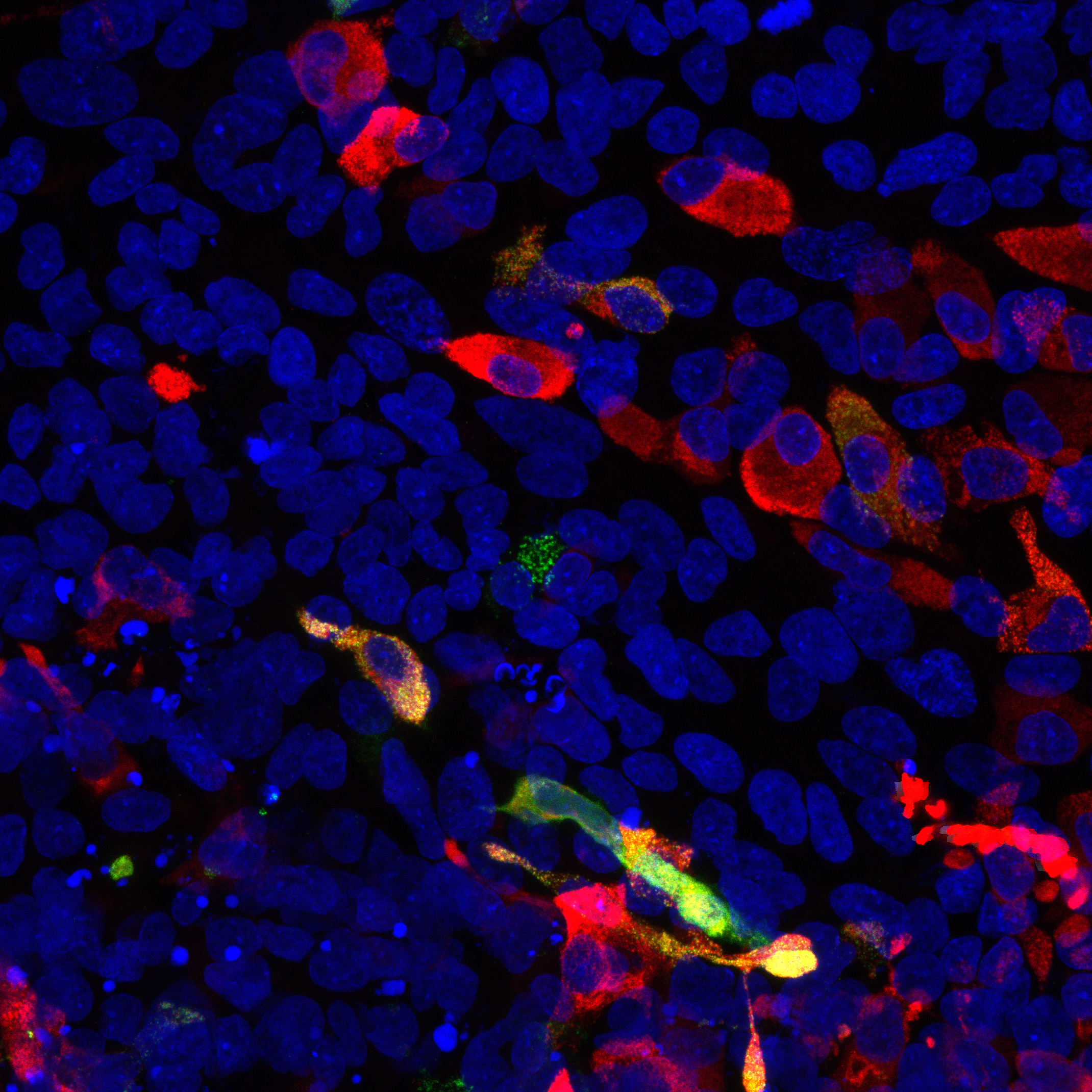

Supplement: Supplementary file 2 — Source data Fig. 1 [file 44319_2025_610_MOESM2_ESM.zip › Figure 1/1A/Fig1A_MIP_LN511.png]

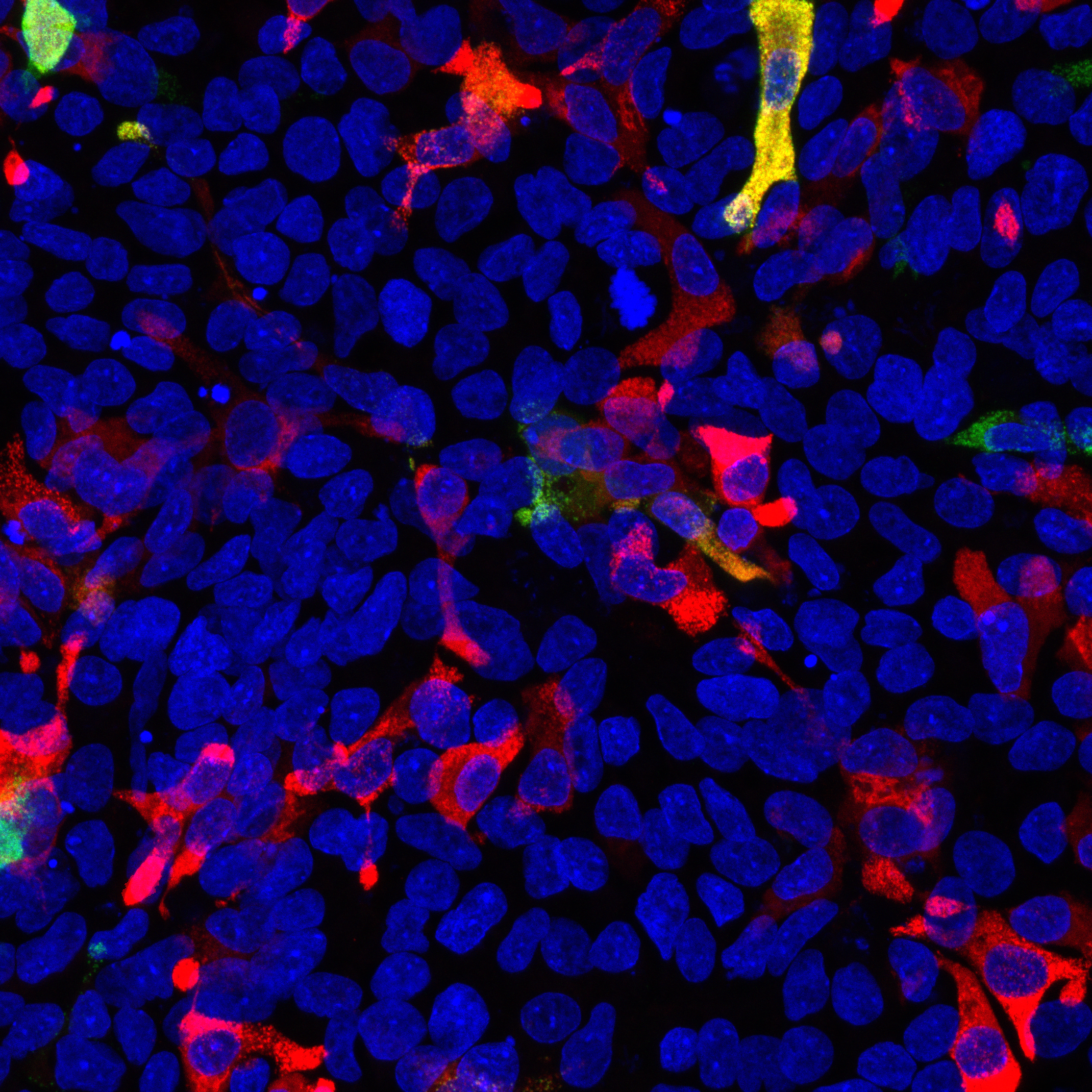

Supplement: Supplementary file 2 — Source data Fig. 1 [file 44319_2025_610_MOESM2_ESM.zip › Figure 1/1A/Fig1A_MIP_LN521.png]

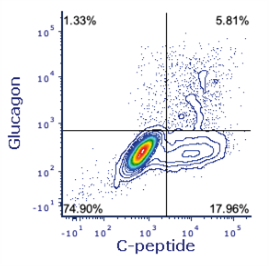

Supplement: Supplementary file 2 — Source data Fig. 1 [file 44319_2025_610_MOESM2_ESM.zip › Figure 1/1C/Fig1C_flowCytometry_FN.png]

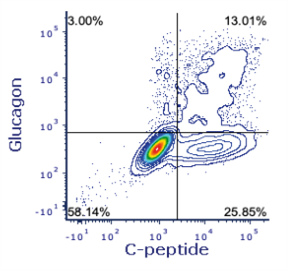

Supplement: Supplementary file 2 — Source data Fig. 1 [file 44319_2025_610_MOESM2_ESM.zip › Figure 1/1C/Fig1C_flowCytometry_LN411.png]

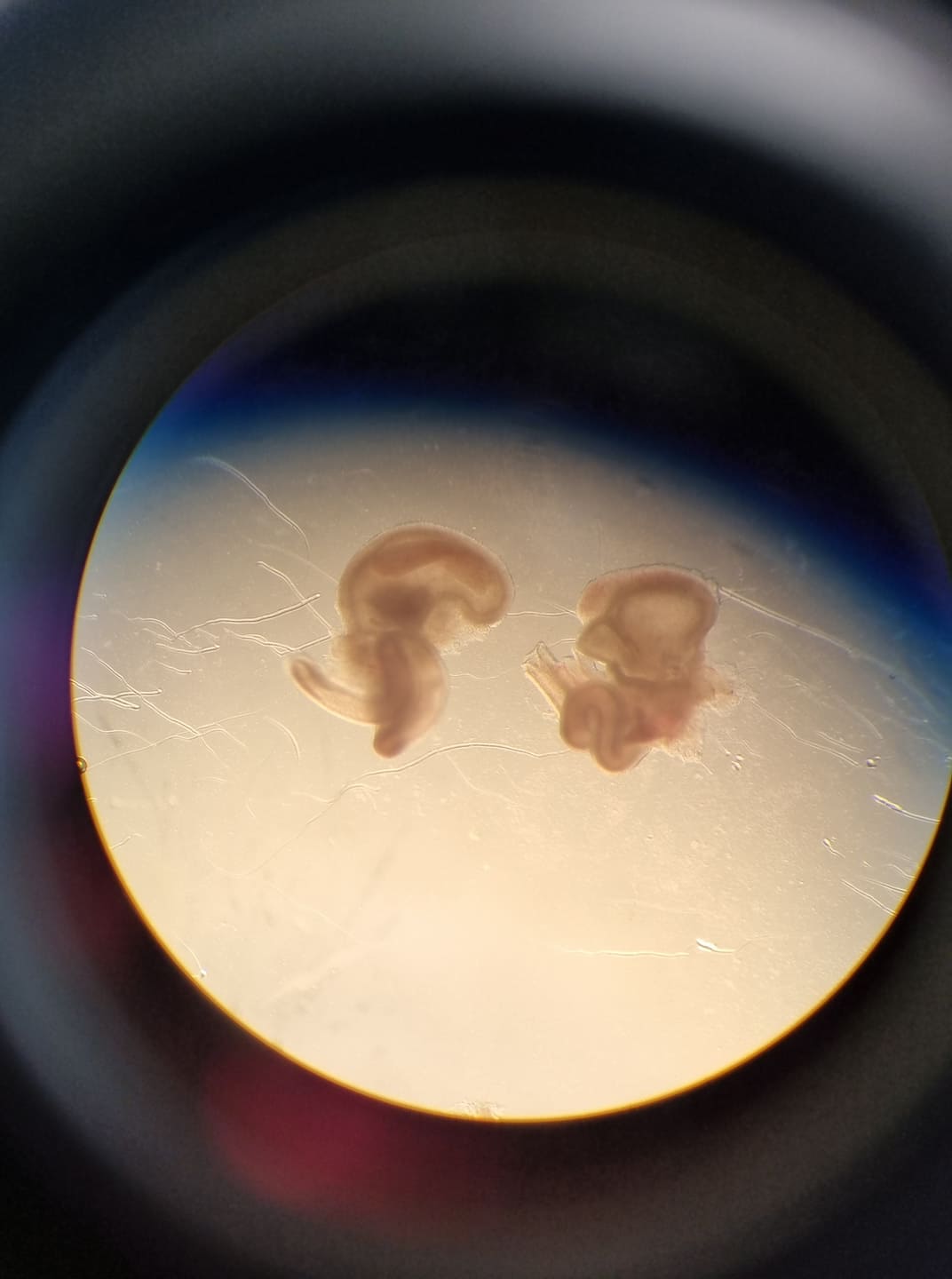

Supplement: Supplementary file 4 — Source data Fig. 3 [file 44319_2025_610_MOESM4_ESM.zip › Figure 3/3B/Fig3B_micrograph_dissectedIntestine.jpg]

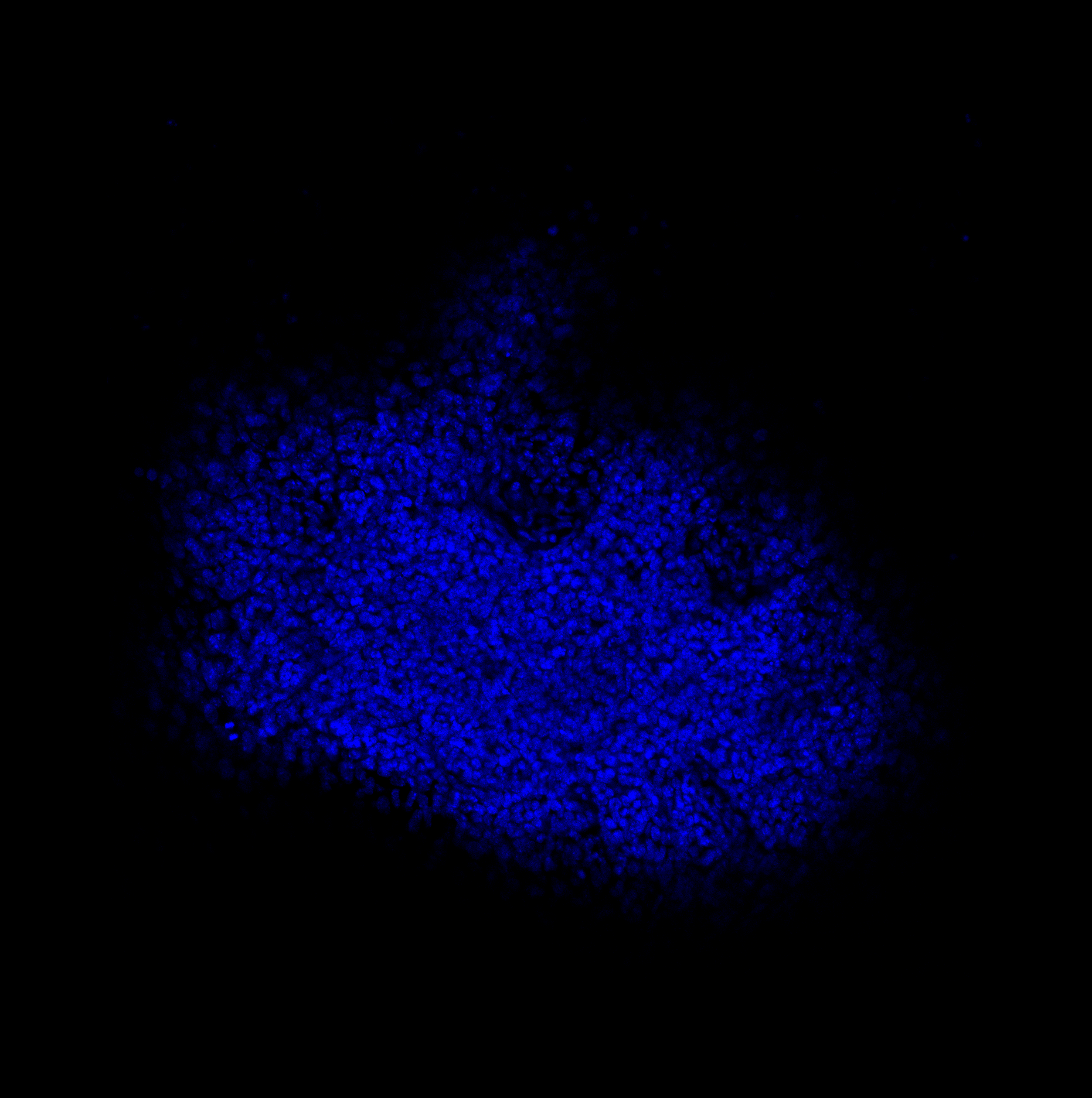

Supplement: Supplementary file 4 — Source data Fig. 3 [file 44319_2025_610_MOESM4_ESM.zip › Figure 3/3C/Fig3B_MIP_DAPI_dKO.png]

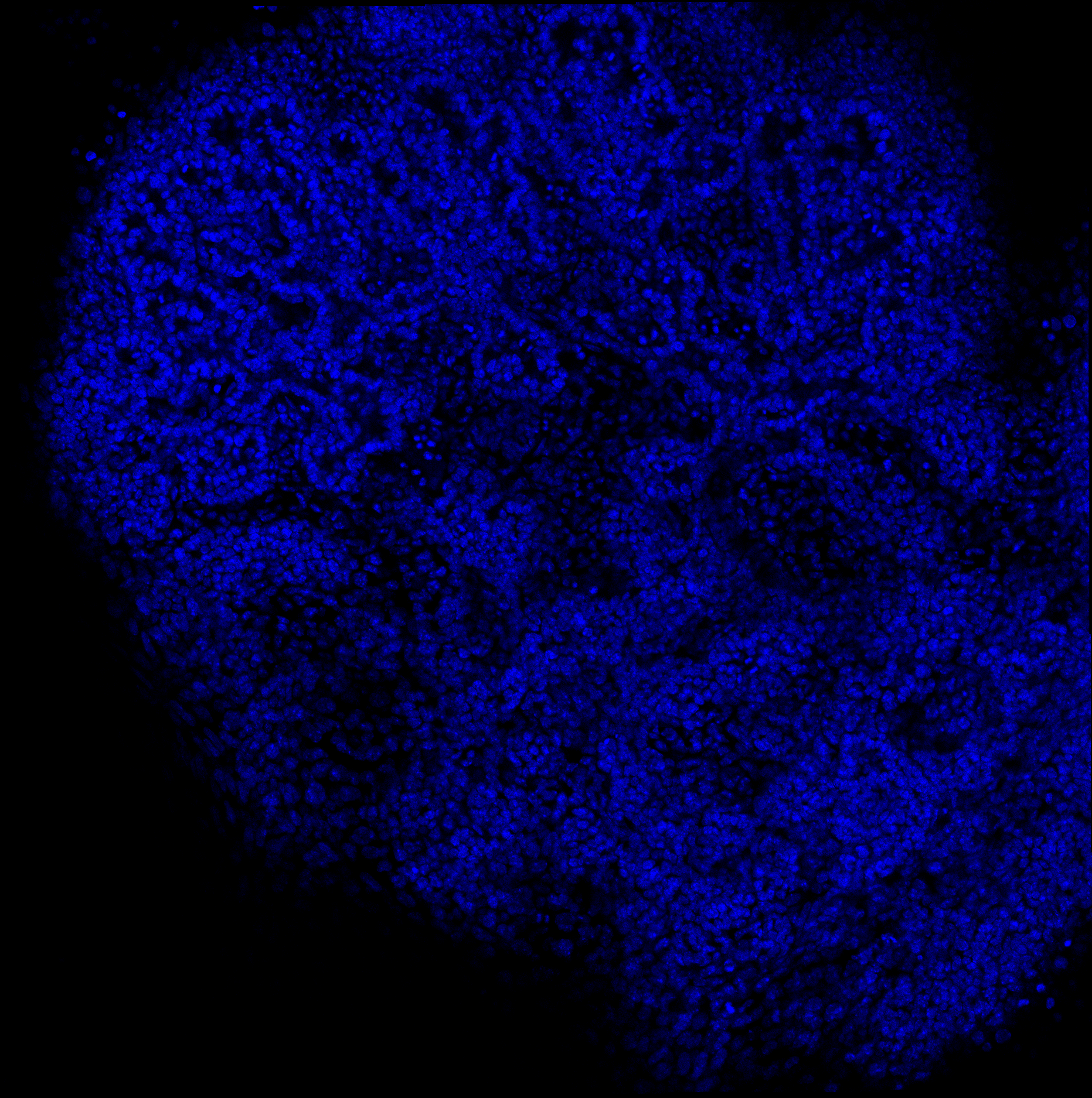

Supplement: Supplementary file 4 — Source data Fig. 3 [file 44319_2025_610_MOESM4_ESM.zip › Figure 3/3C/Fig3B_MIP_DAPI_WT.png]

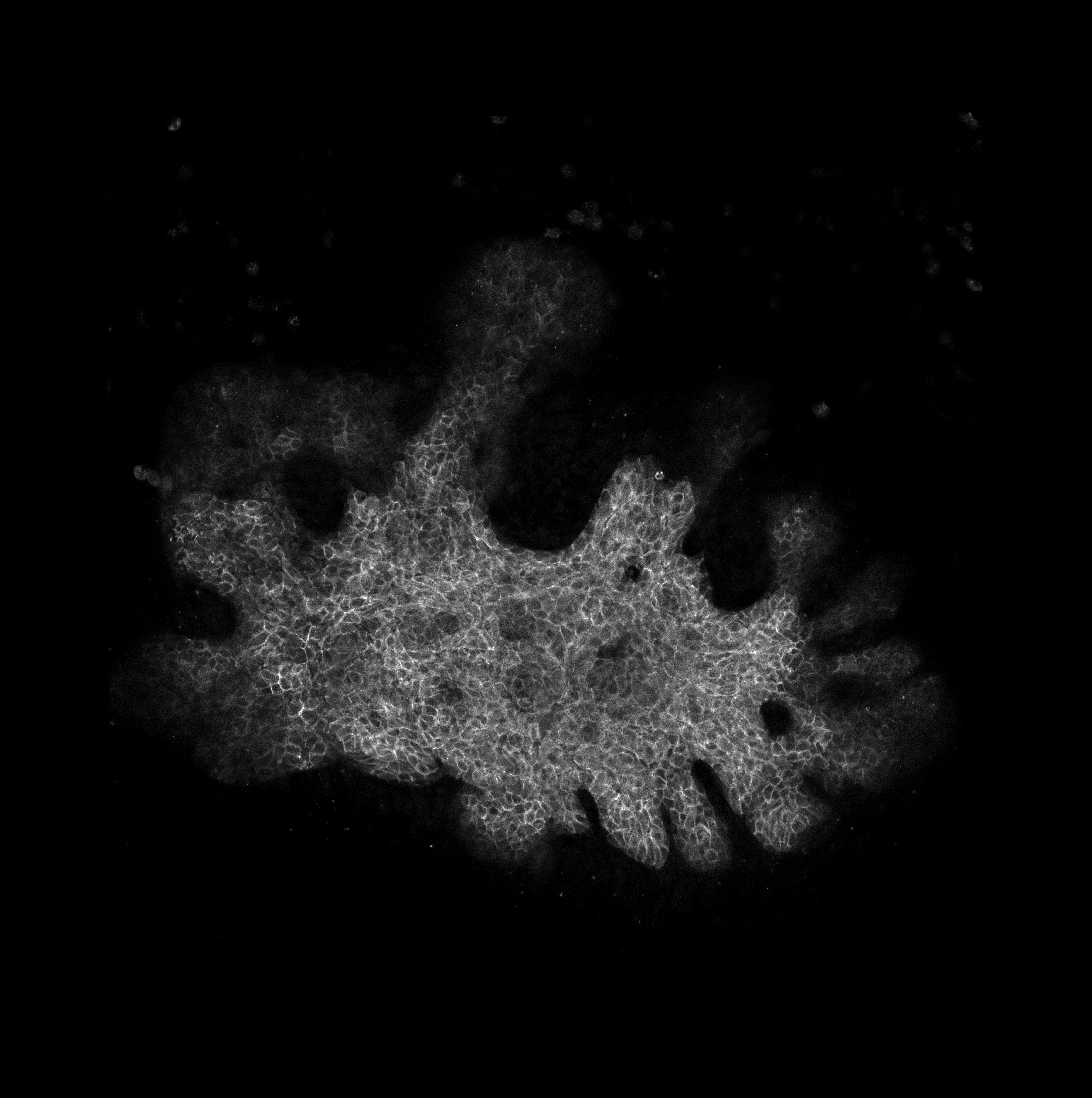

Supplement: Supplementary file 4 — Source data Fig. 3 [file 44319_2025_610_MOESM4_ESM.zip › Figure 3/3C/Fig3B_MIP_Ecad_dKO.png]

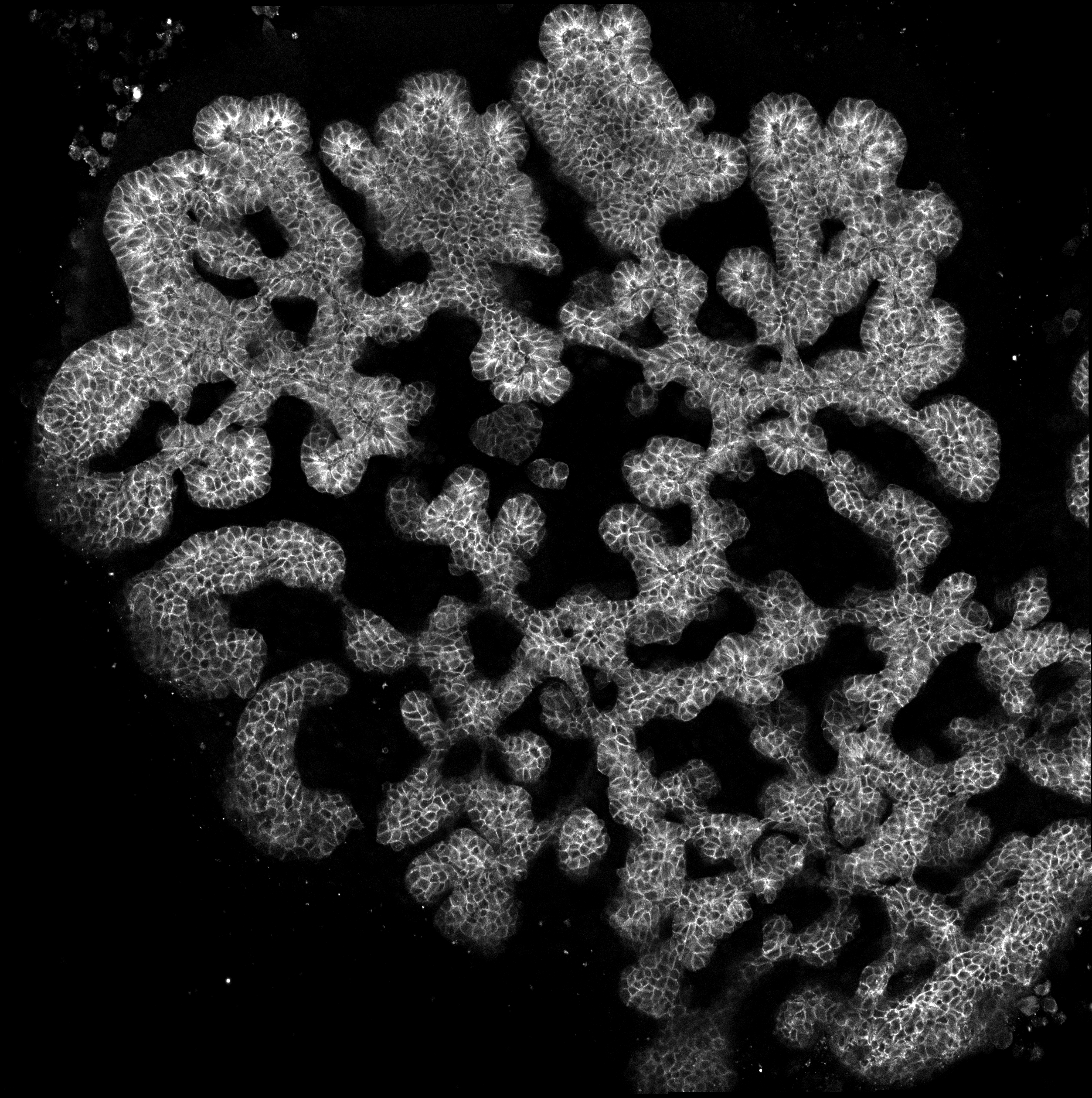

Supplement: Supplementary file 4 — Source data Fig. 3 [file 44319_2025_610_MOESM4_ESM.zip › Figure 3/3C/Fig3B_MIP_Ecad_WT.png]

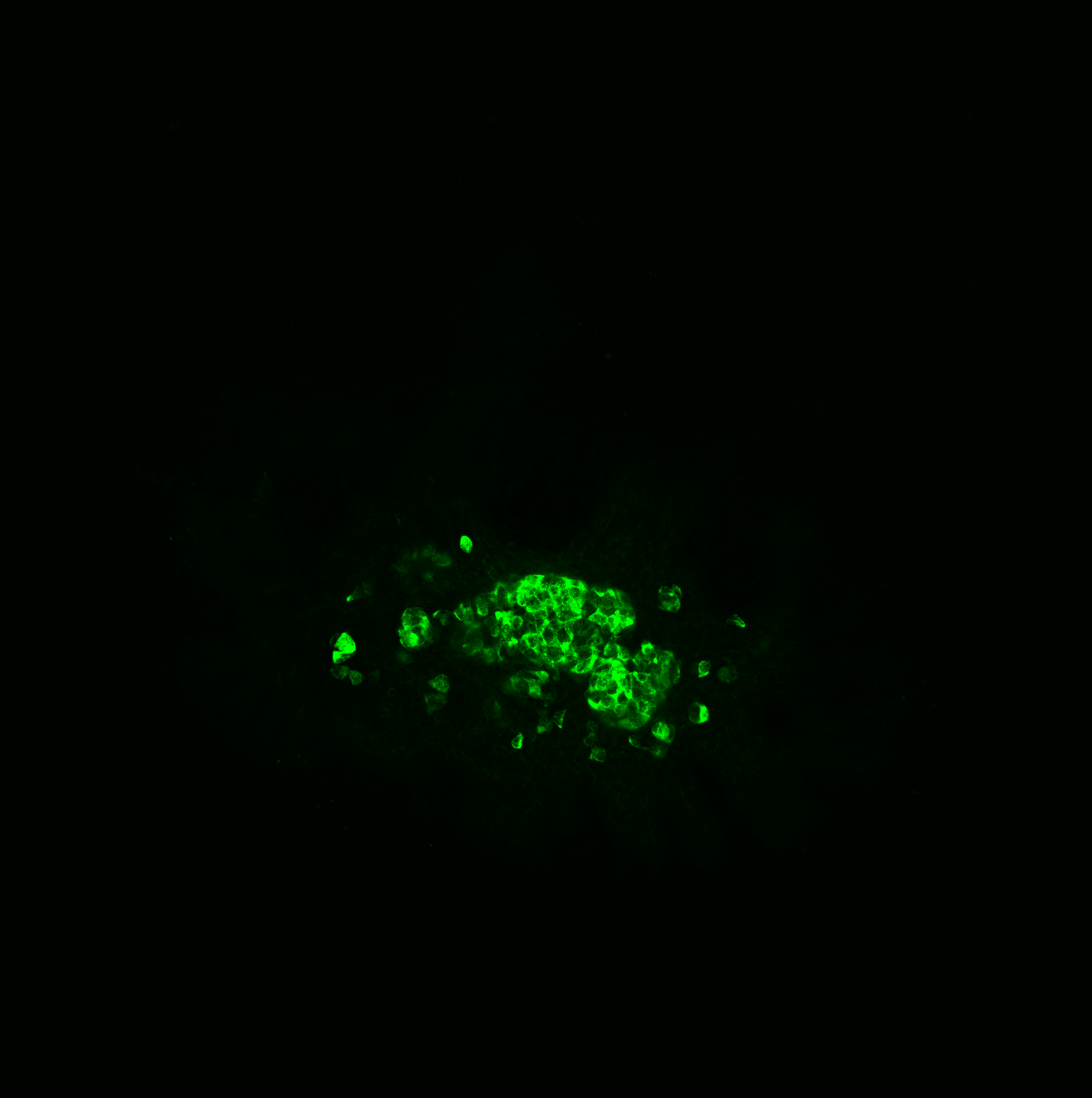

Supplement: Supplementary file 4 — Source data Fig. 3 [file 44319_2025_610_MOESM4_ESM.zip › Figure 3/3C/Fig3B_MIP_Gcg_dKO.png]

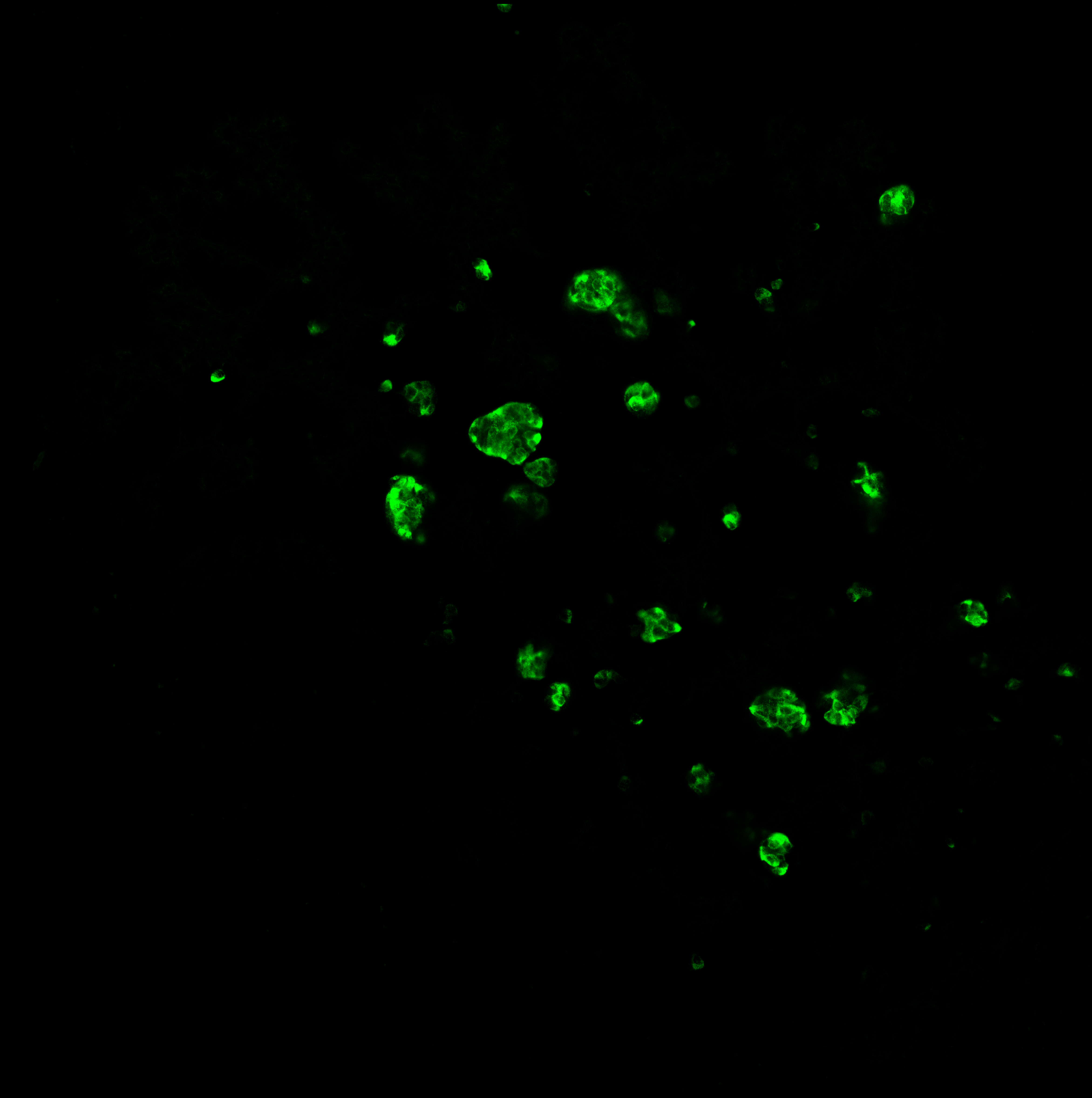

Supplement: Supplementary file 4 — Source data Fig. 3 [file 44319_2025_610_MOESM4_ESM.zip › Figure 3/3C/Fig3B_MIP_Gcg_WT.png]

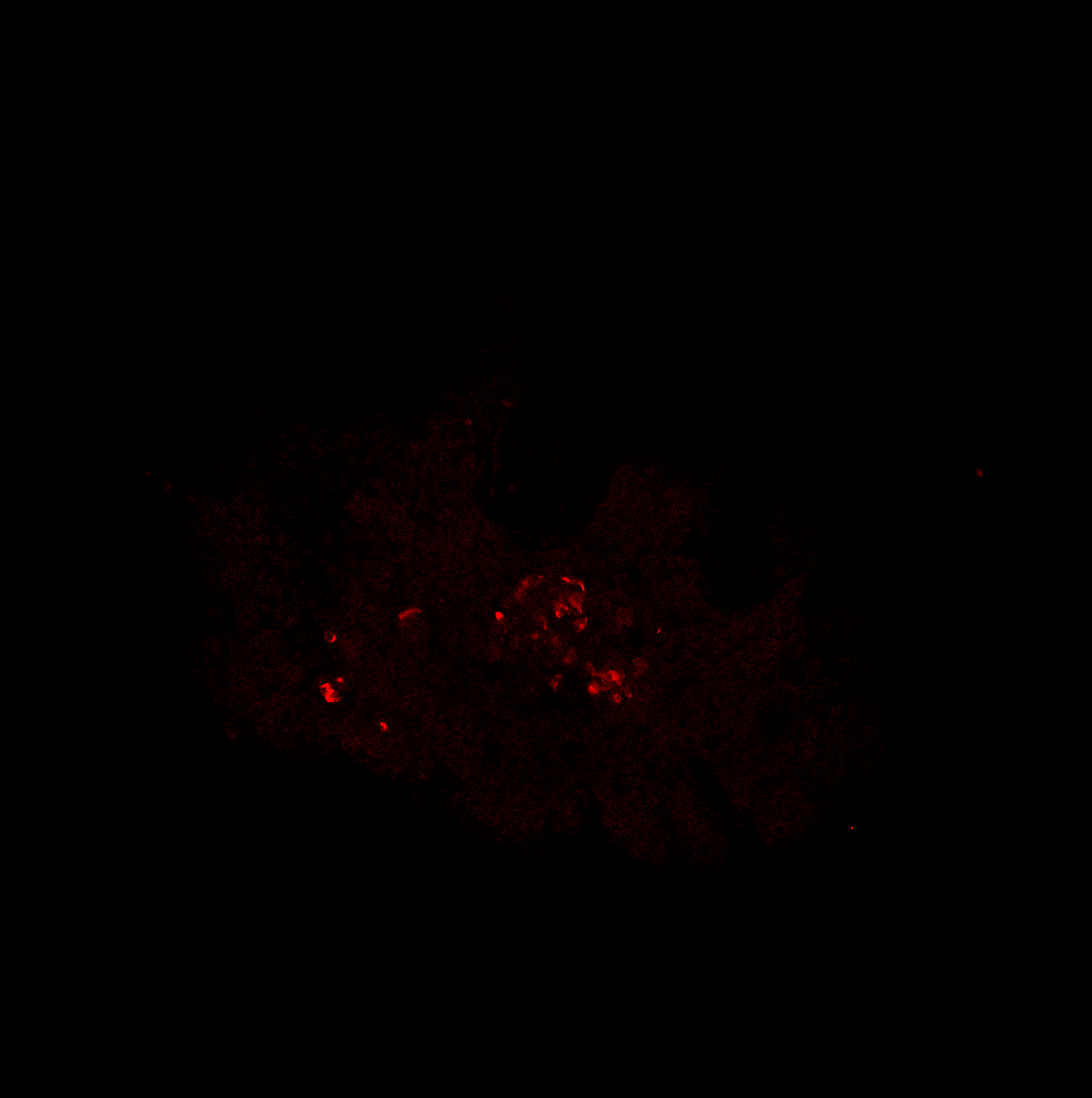

Supplement: Supplementary file 4 — Source data Fig. 3 [file 44319_2025_610_MOESM4_ESM.zip › Figure 3/3C/Fig3B_MIP_Ins_dKO.png]

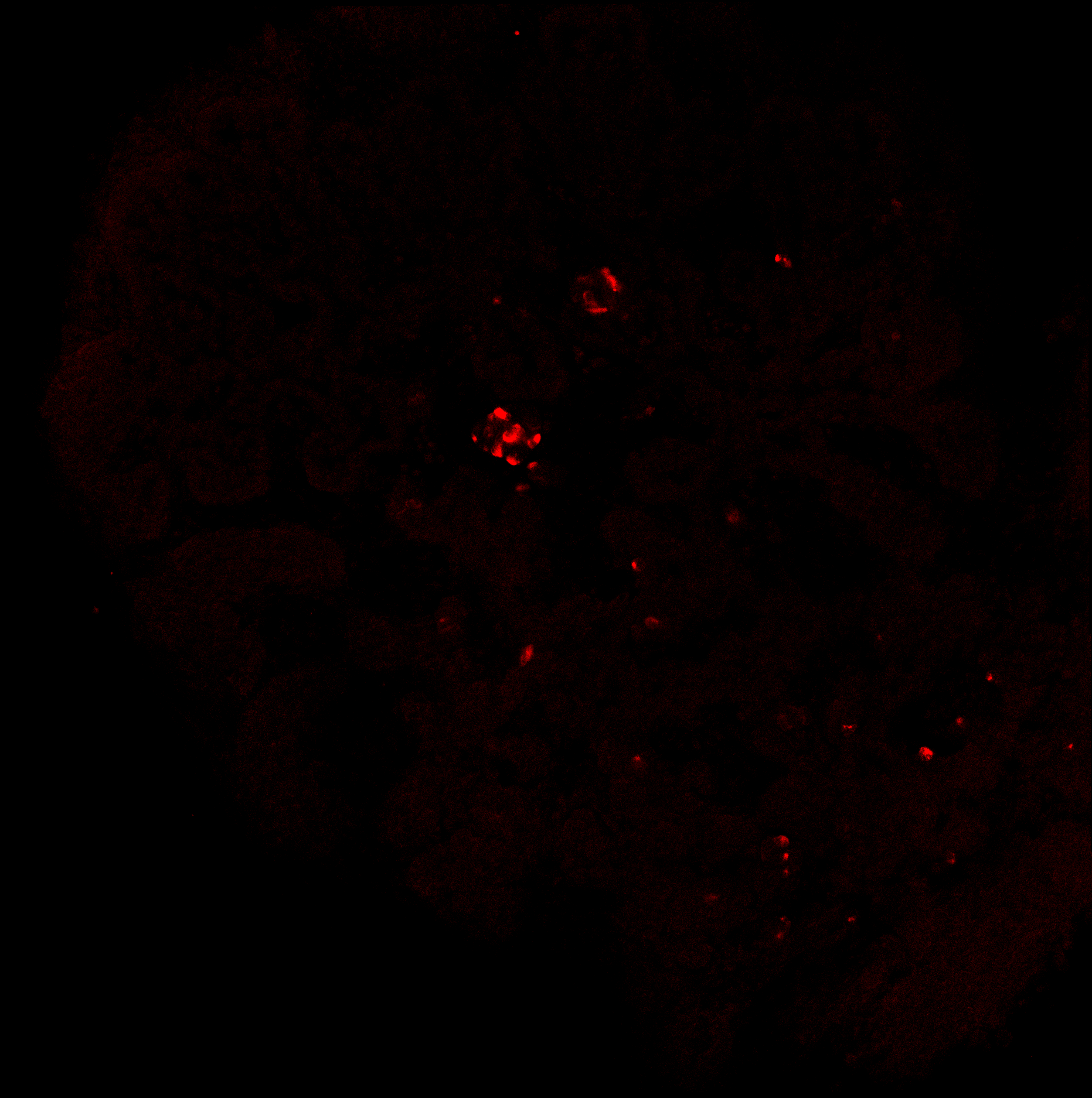

Supplement: Supplementary file 4 — Source data Fig. 3 [file 44319_2025_610_MOESM4_ESM.zip › Figure 3/3C/Fig3B_MIP_Ins_WT.png]

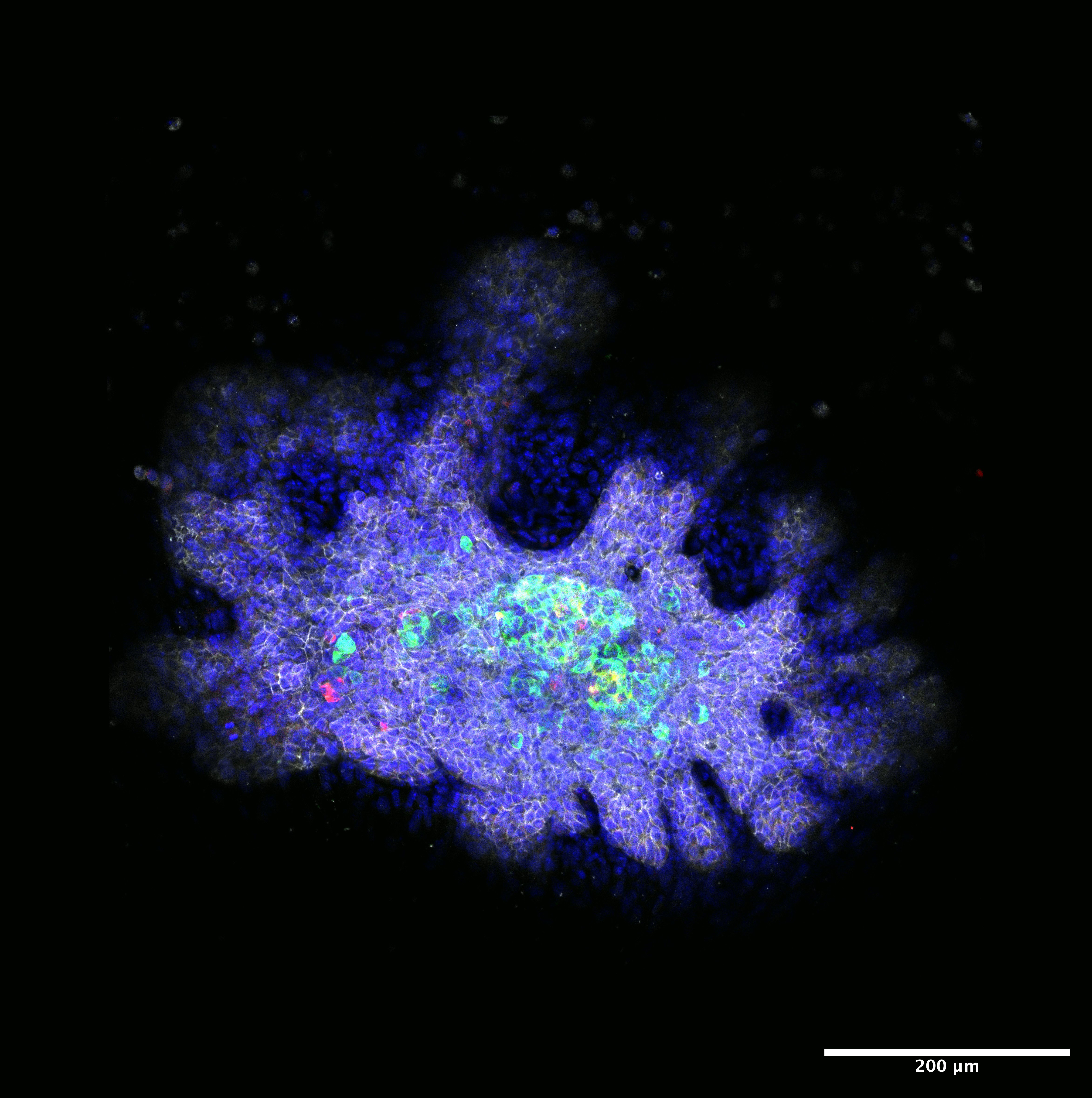

Supplement: Supplementary file 4 — Source data Fig. 3 [file 44319_2025_610_MOESM4_ESM.zip › Figure 3/3C/Fig3B_MIP_Merged_dKO.png]

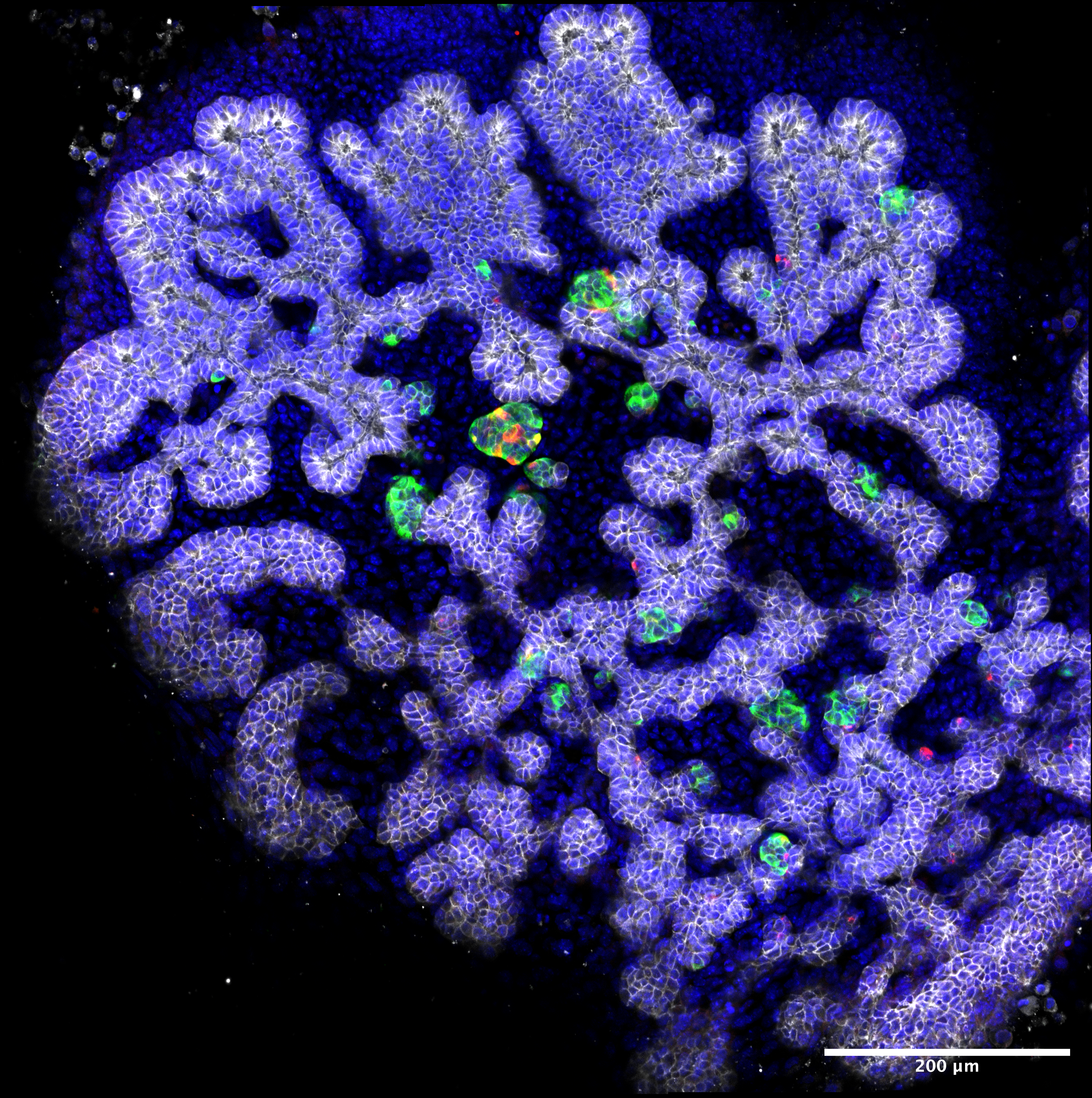

Supplement: Supplementary file 4 — Source data Fig. 3 [file 44319_2025_610_MOESM4_ESM.zip › Figure 3/3C/Fig3B_MIP_Merged_WT.png]

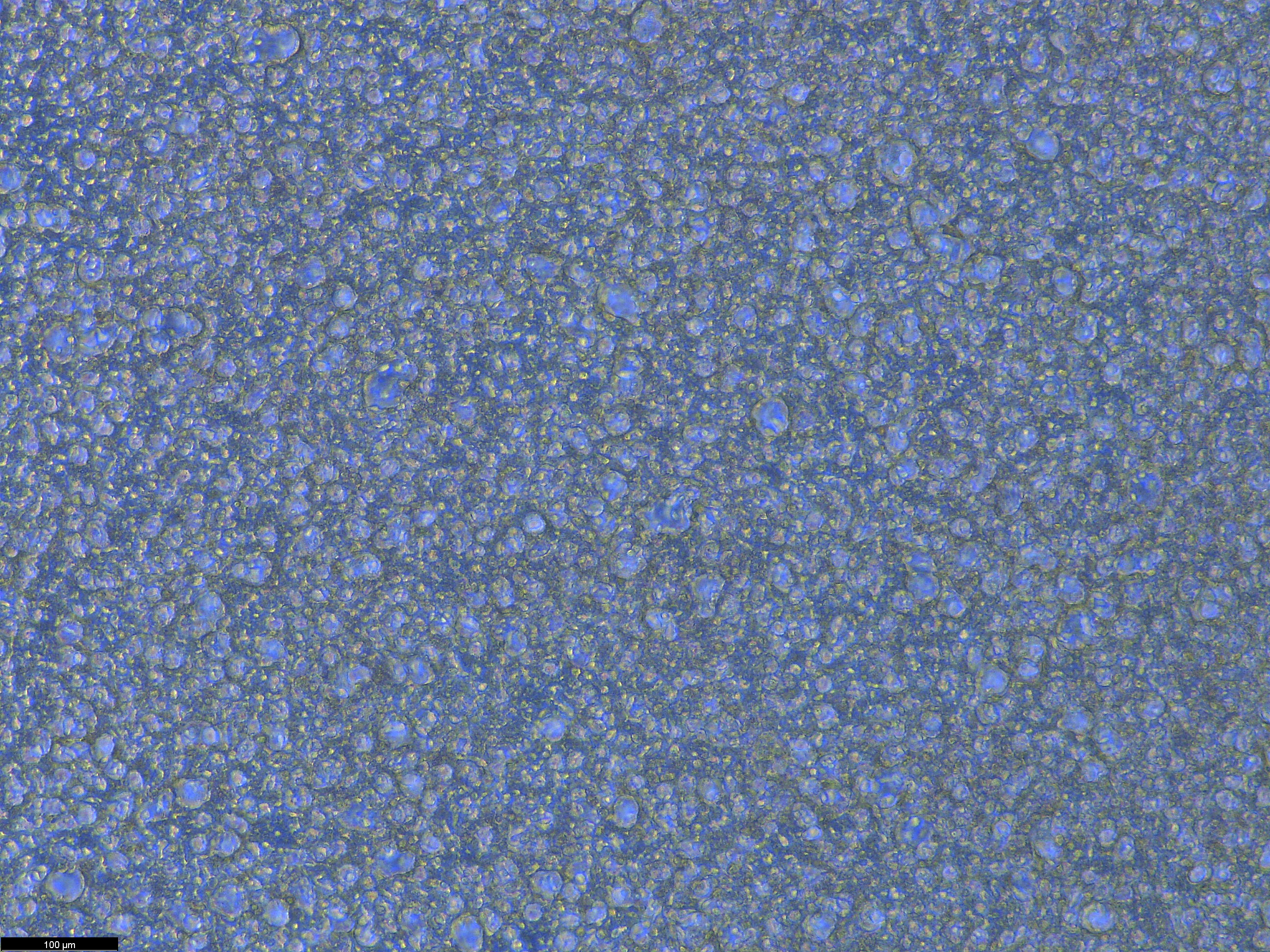

Supplement: Supplementary file 5 — Source data Fig. 4 [file 44319_2025_610_MOESM5_ESM.zip › Figure 4/4D/Fig4D_LN411.png]

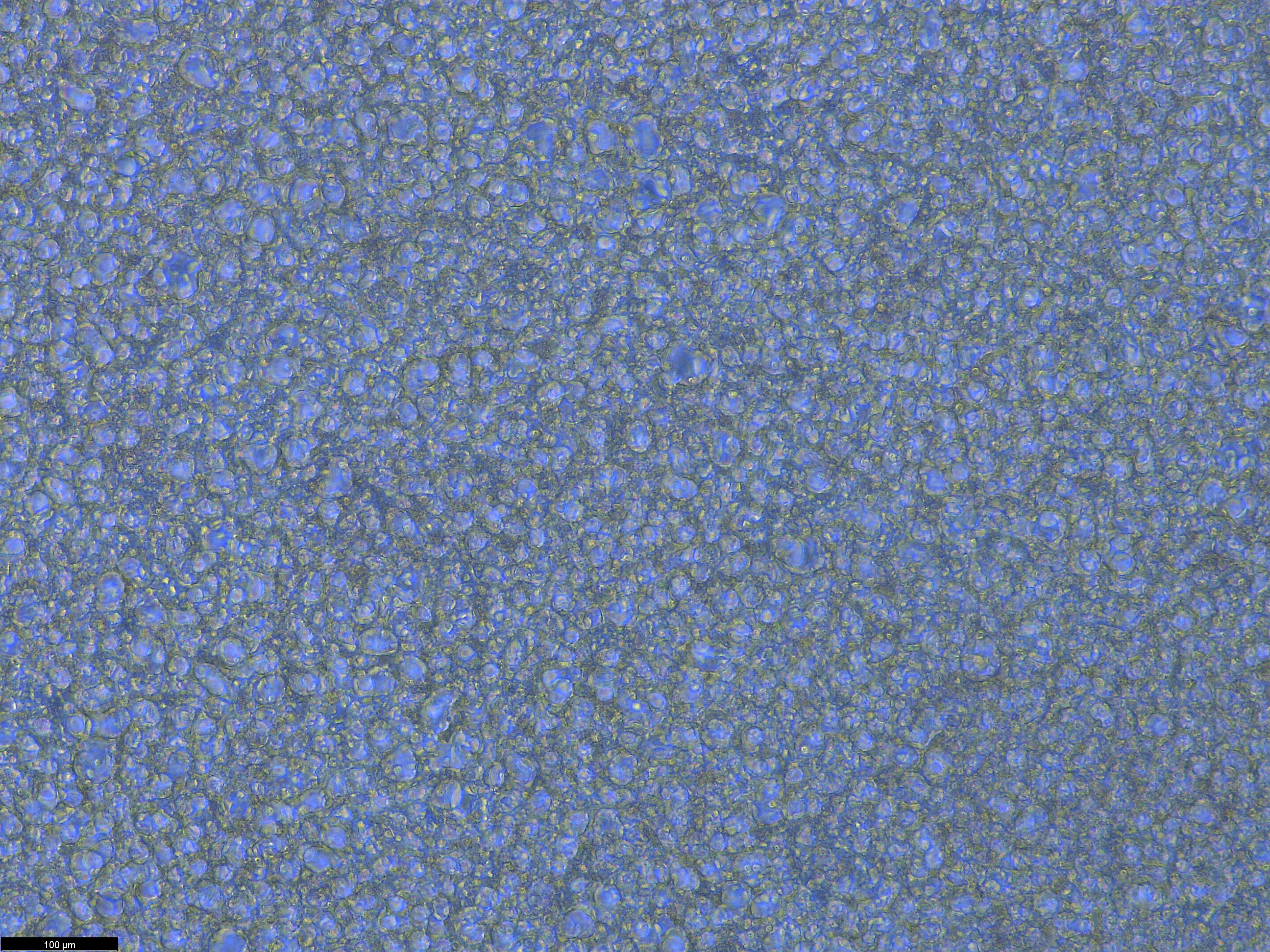

Supplement: Supplementary file 5 — Source data Fig. 4 [file 44319_2025_610_MOESM5_ESM.zip › Figure 4/4D/Fig4D_LN411DCA.png]

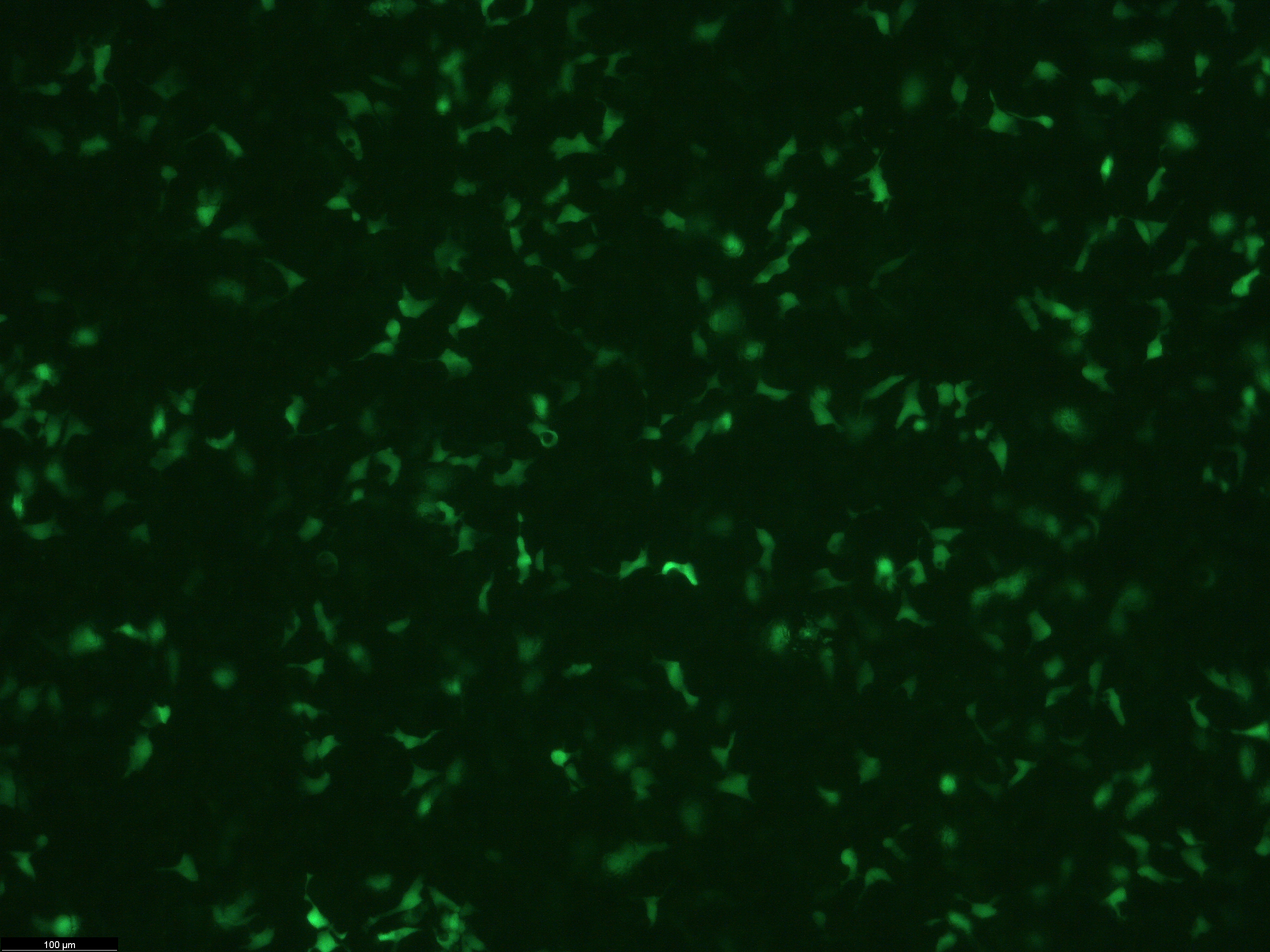

Supplement: Supplementary file 5 — Source data Fig. 4 [file 44319_2025_610_MOESM5_ESM.zip › Figure 4/4D/Fig4D_LN411DCAGFP.png]

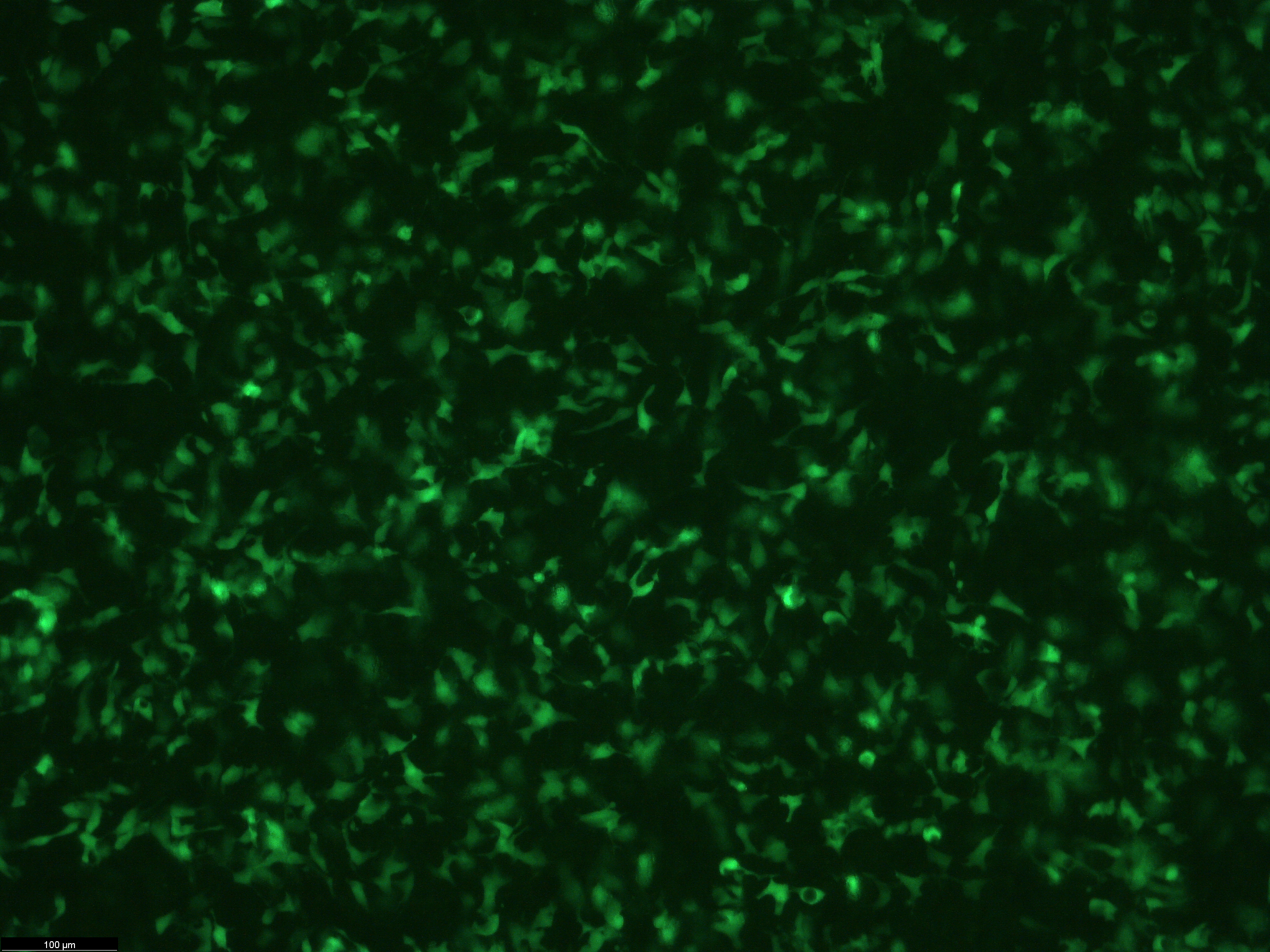

Supplement: Supplementary file 5 — Source data Fig. 4 [file 44319_2025_610_MOESM5_ESM.zip › Figure 4/4D/Fig4D_LN411GFP.png]

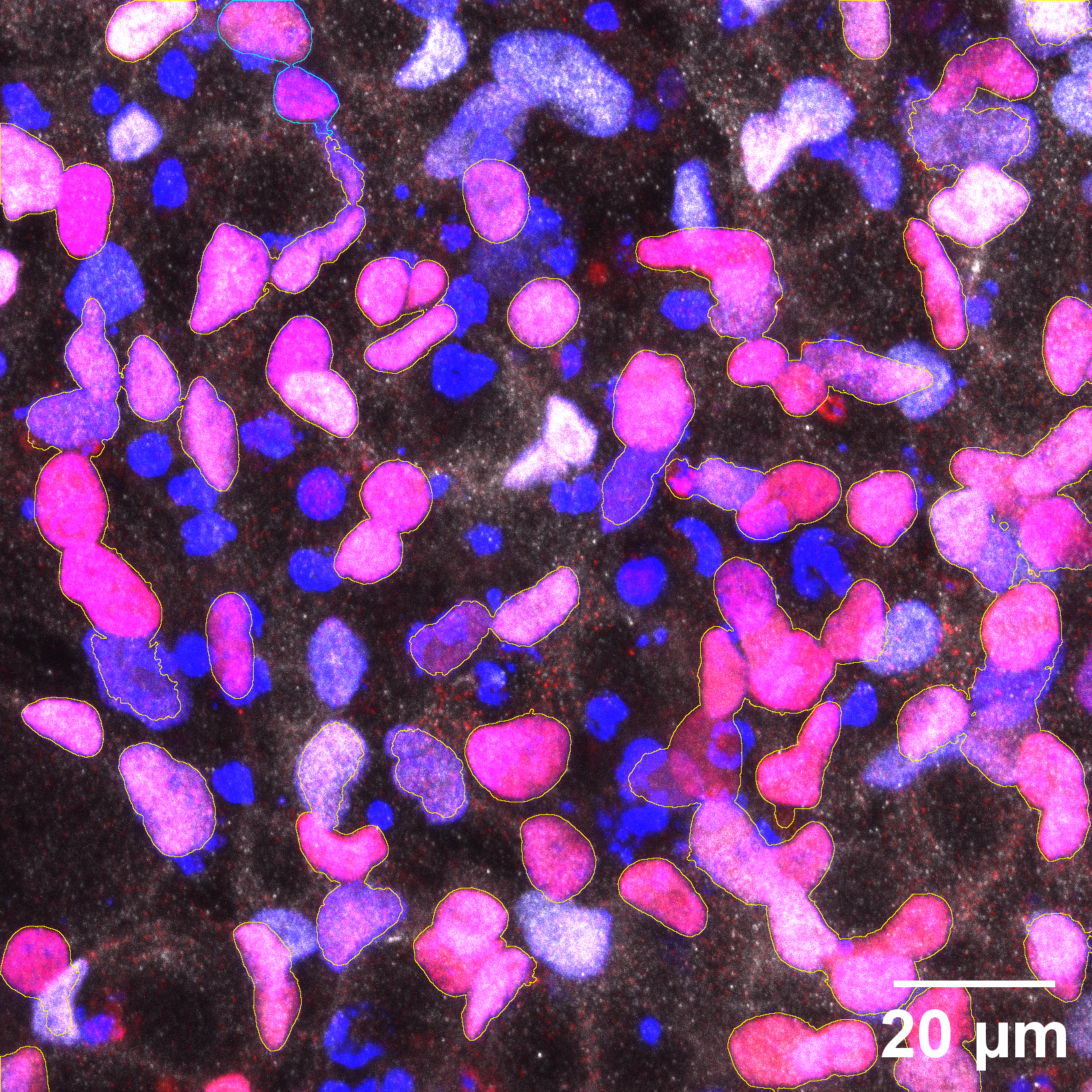

Supplement: Supplementary file 6 — Source data Fig. 5 [file 44319_2025_610_MOESM6_ESM.zip › Figure 5/5A/Fig5A_MIP_LN411DCA_combined.png]

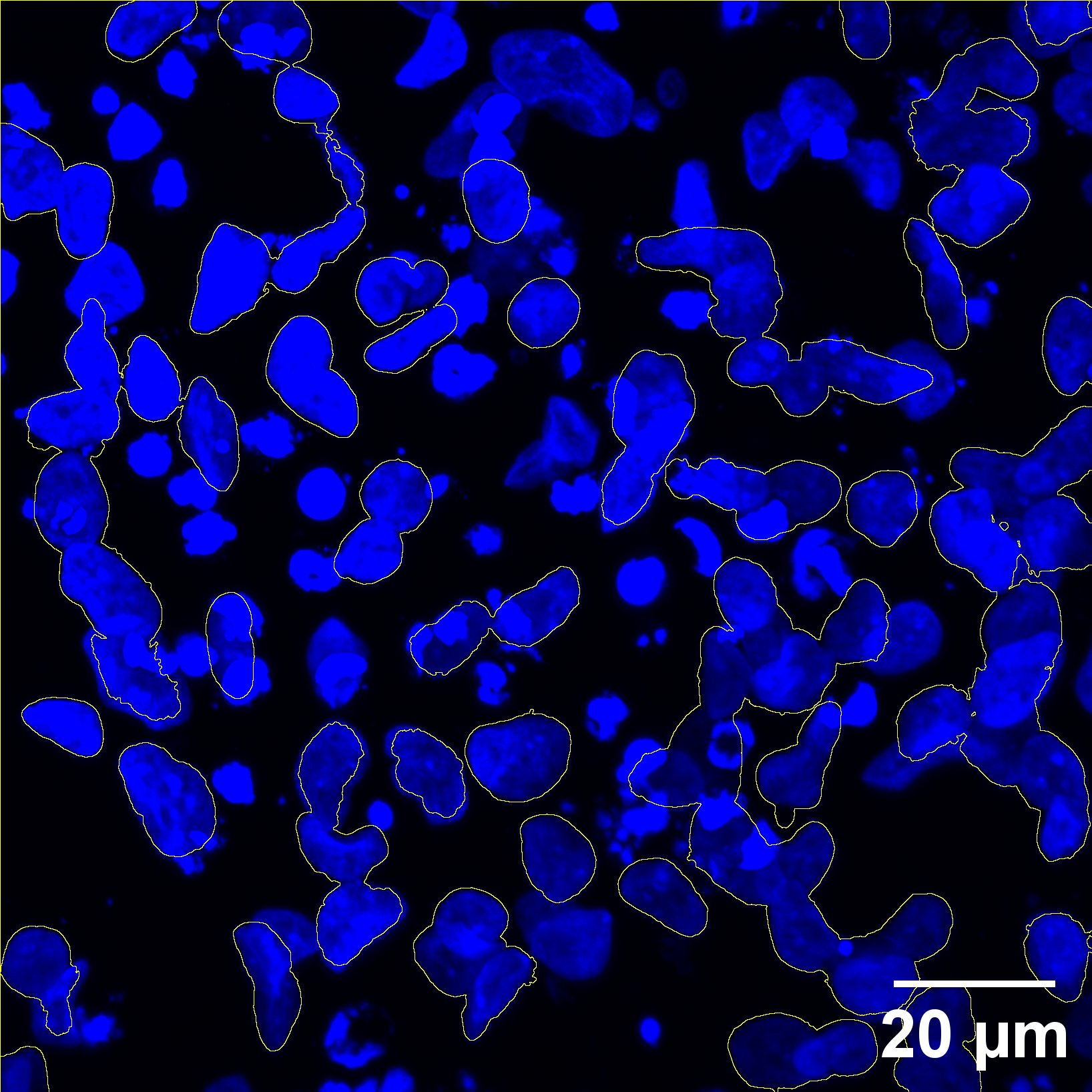

Supplement: Supplementary file 6 — Source data Fig. 5 [file 44319_2025_610_MOESM6_ESM.zip › Figure 5/5A/Fig5A_MIP_LN411DCA_DAPI.png]

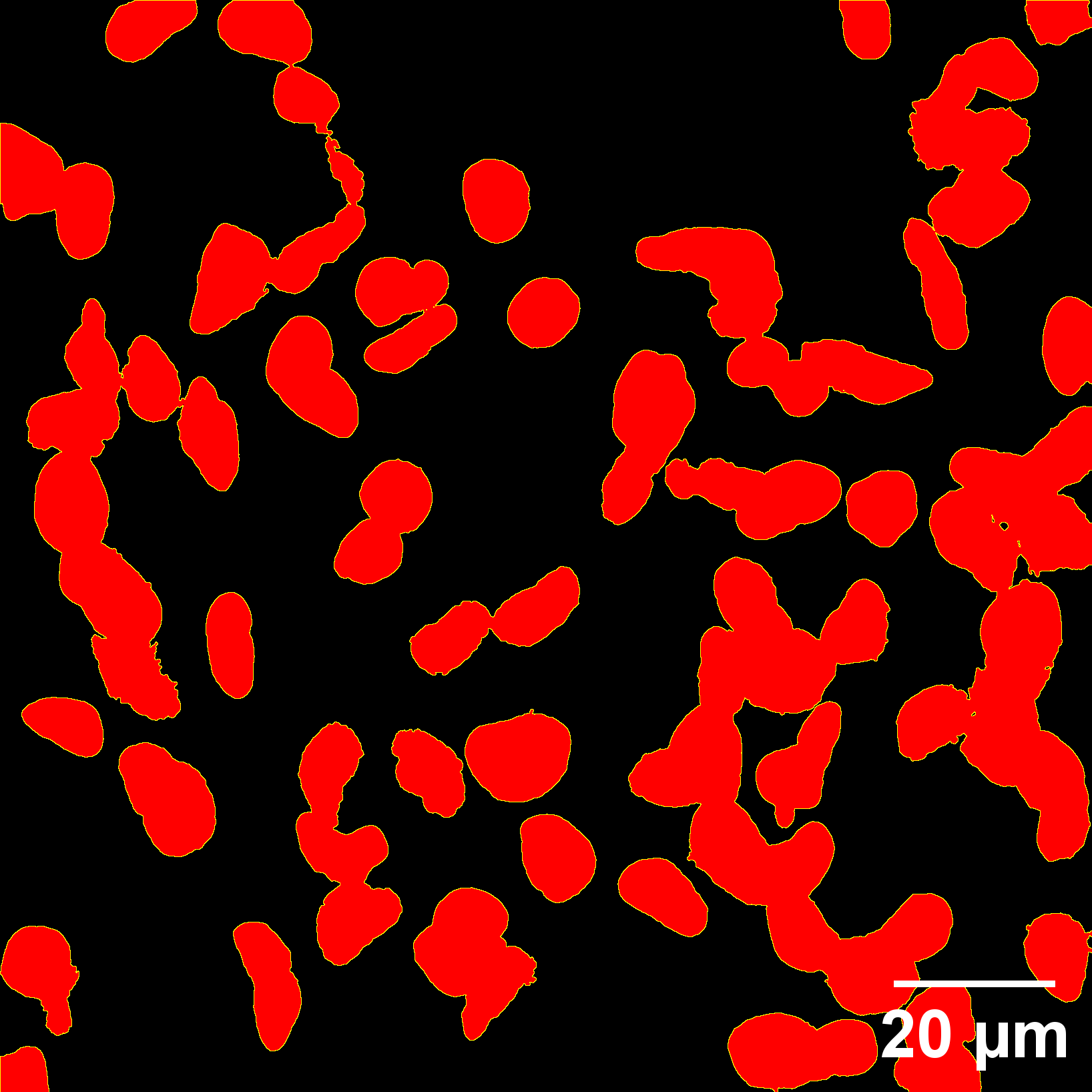

Supplement: Supplementary file 6 — Source data Fig. 5 [file 44319_2025_610_MOESM6_ESM.zip › Figure 5/5A/Fig5A_MIP_LN411DCA_Mask.png]

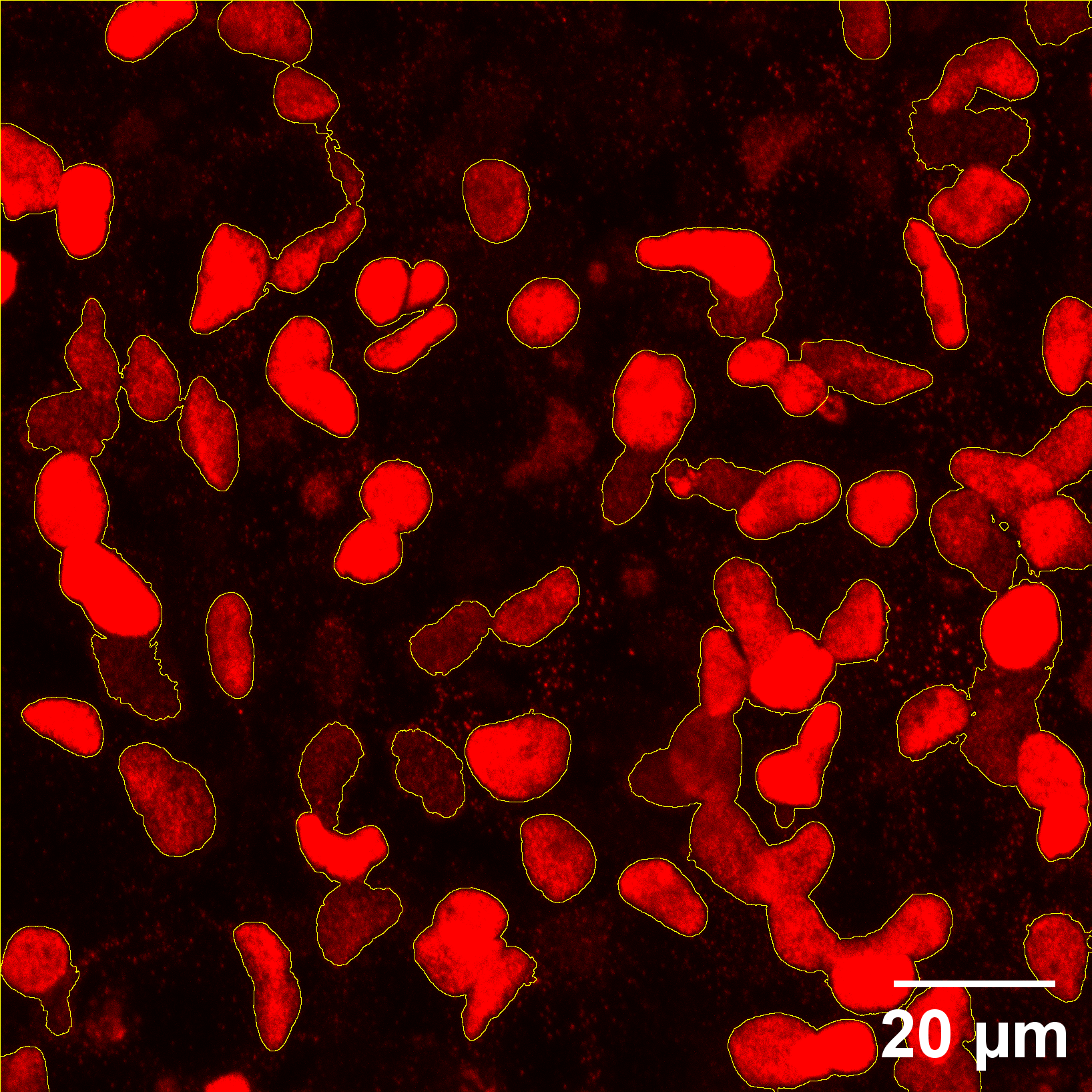

Supplement: Supplementary file 6 — Source data Fig. 5 [file 44319_2025_610_MOESM6_ESM.zip › Figure 5/5A/Fig5A_MIP_LN411DCA_NKX61.png]

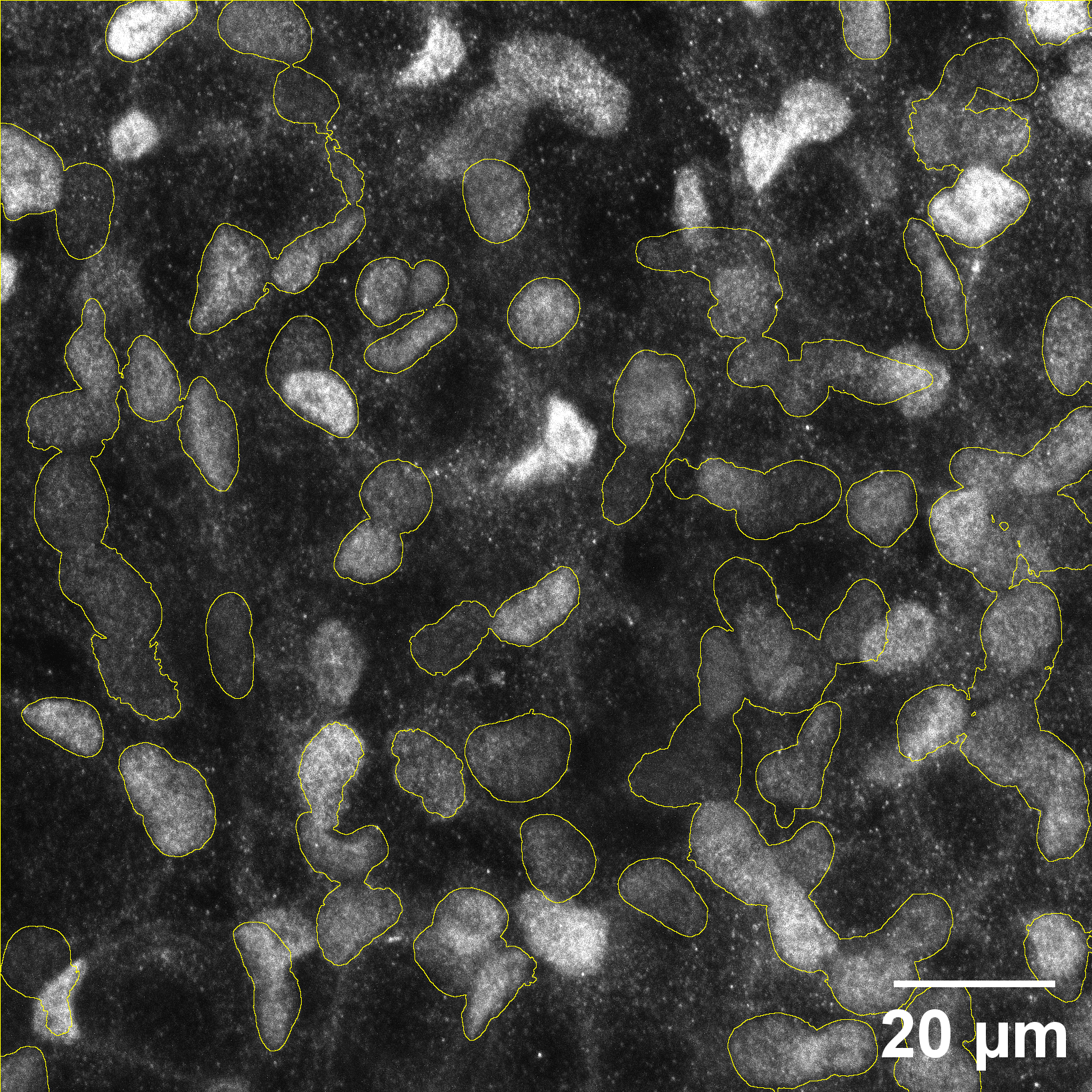

Supplement: Supplementary file 6 — Source data Fig. 5 [file 44319_2025_610_MOESM6_ESM.zip › Figure 5/5A/Fig5A_MIP_LN411DCA_YAP.png]

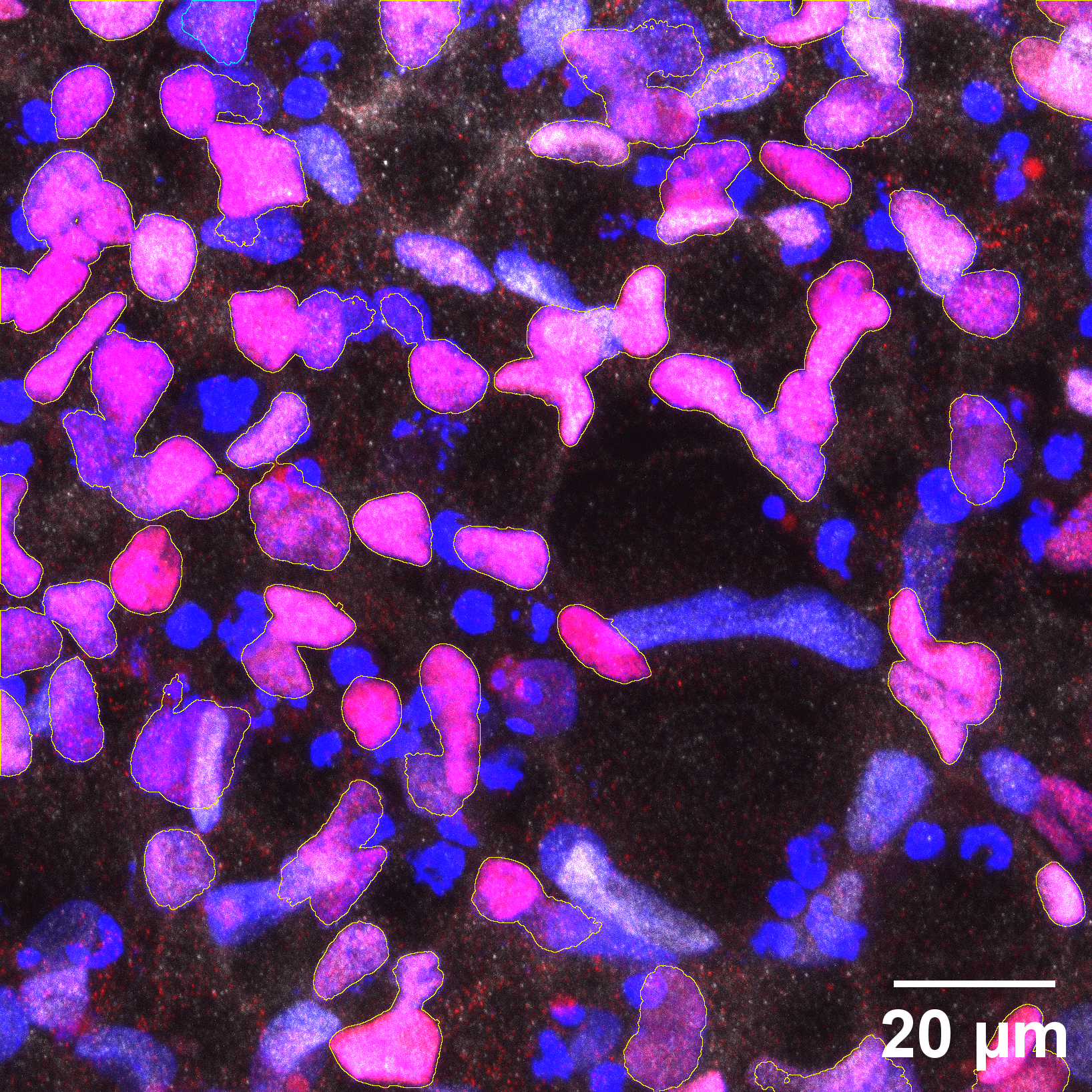

Supplement: Supplementary file 6 — Source data Fig. 5 [file 44319_2025_610_MOESM6_ESM.zip › Figure 5/5A/Fig5A_MIP_LN411_combined.png]

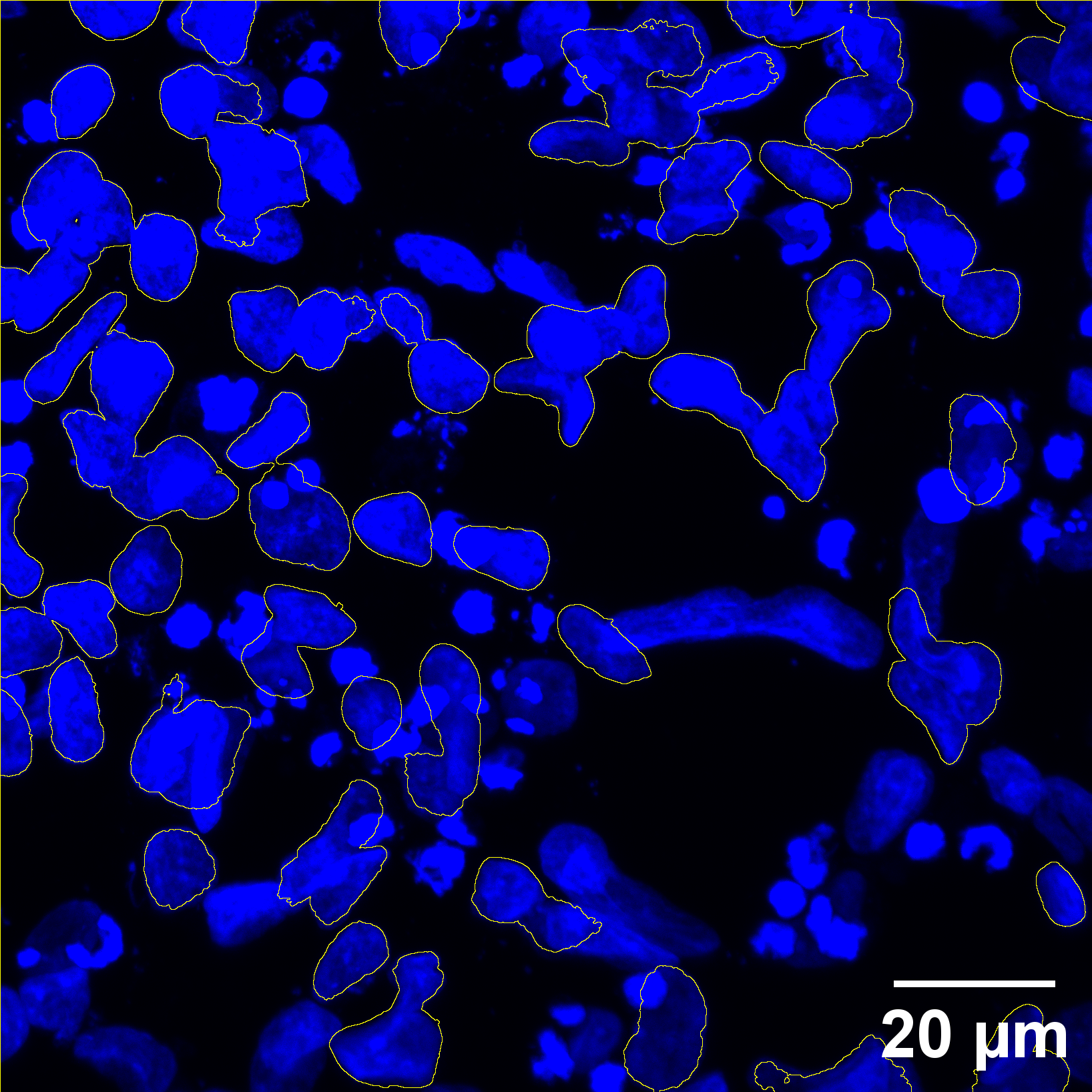

Supplement: Supplementary file 6 — Source data Fig. 5 [file 44319_2025_610_MOESM6_ESM.zip › Figure 5/5A/Fig5A_MIP_LN411_DAPI.png]

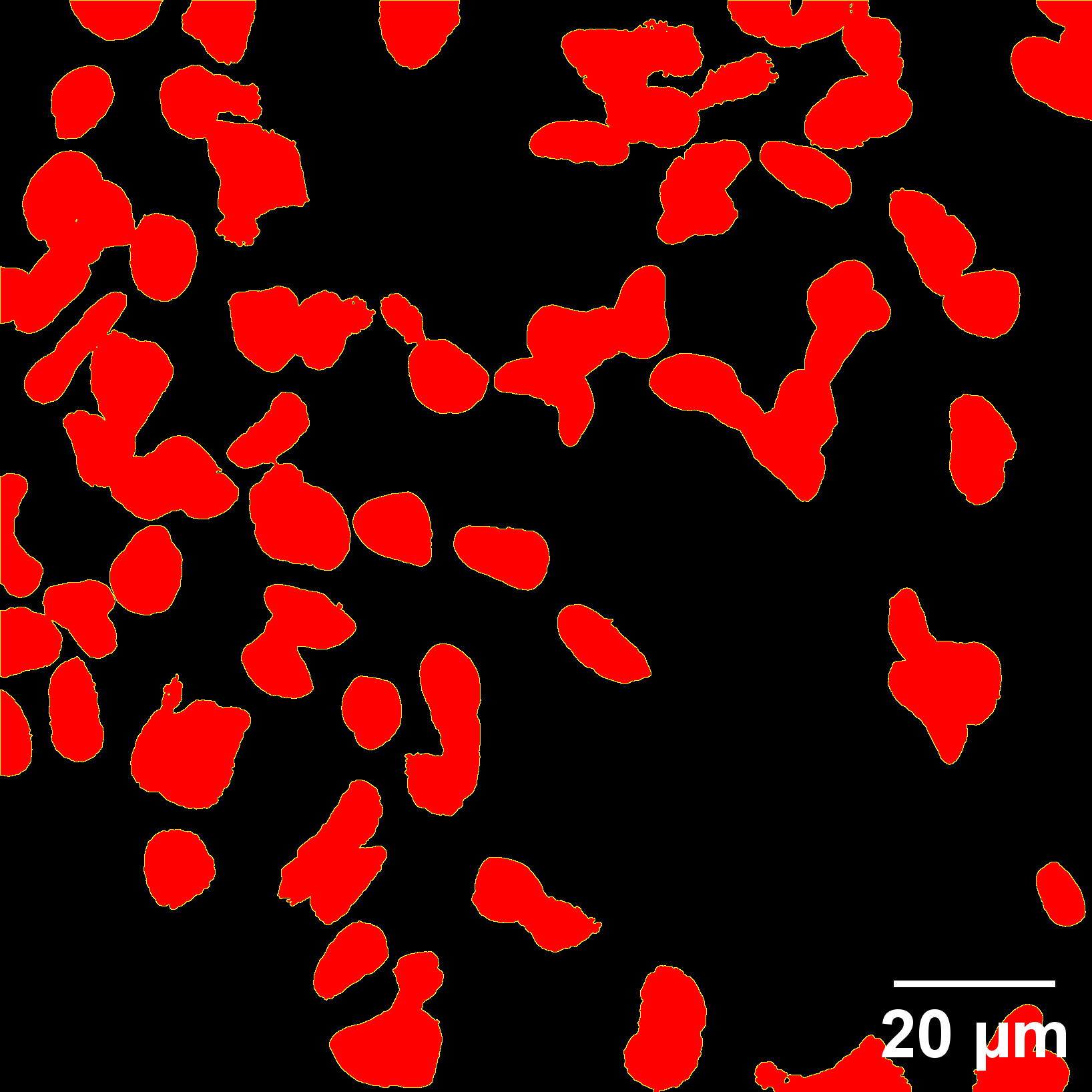

Supplement: Supplementary file 6 — Source data Fig. 5 [file 44319_2025_610_MOESM6_ESM.zip › Figure 5/5A/Fig5A_MIP_LN411_Mask.png]

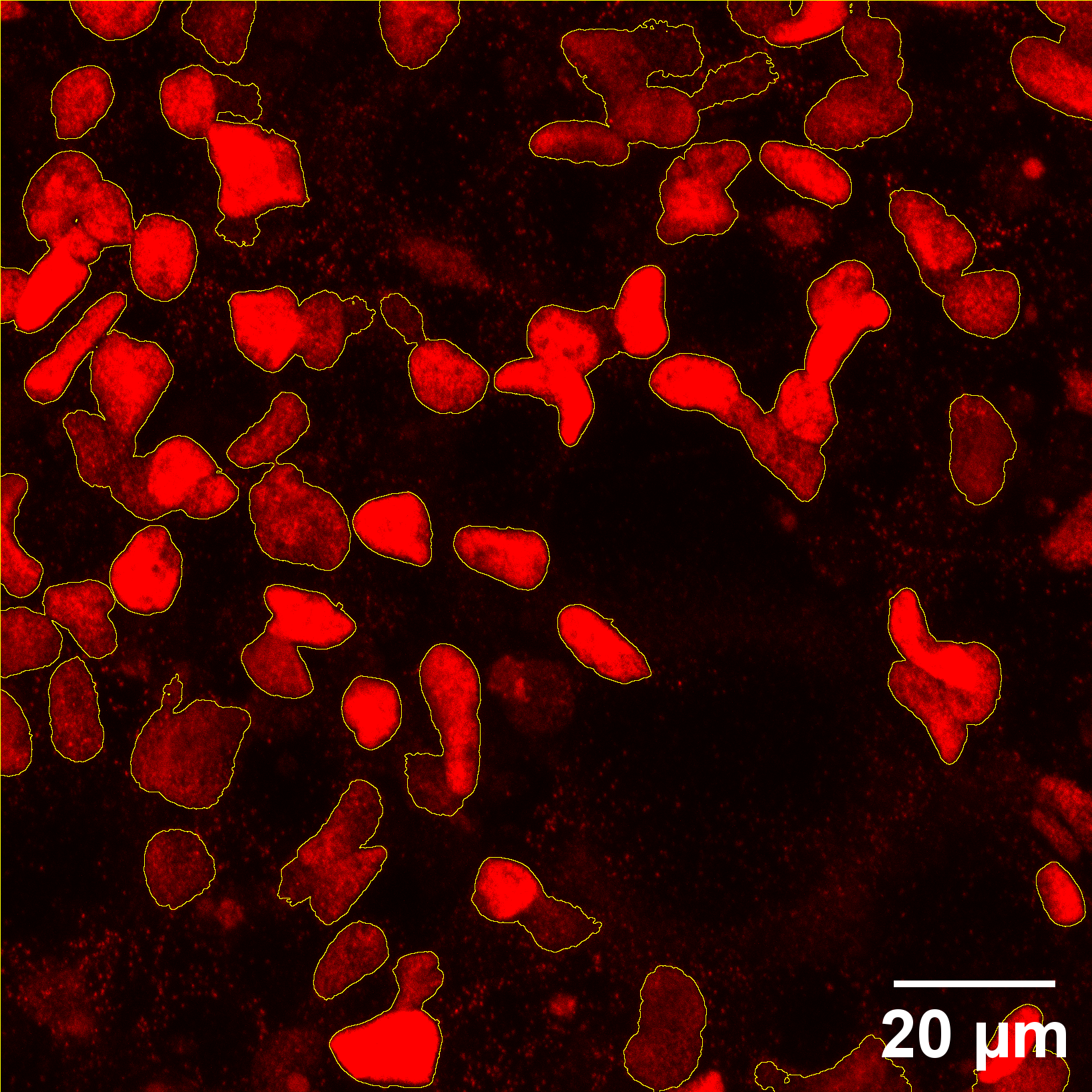

Supplement: Supplementary file 6 — Source data Fig. 5 [file 44319_2025_610_MOESM6_ESM.zip › Figure 5/5A/Fig5A_MIP_LN411_NKX61.png]

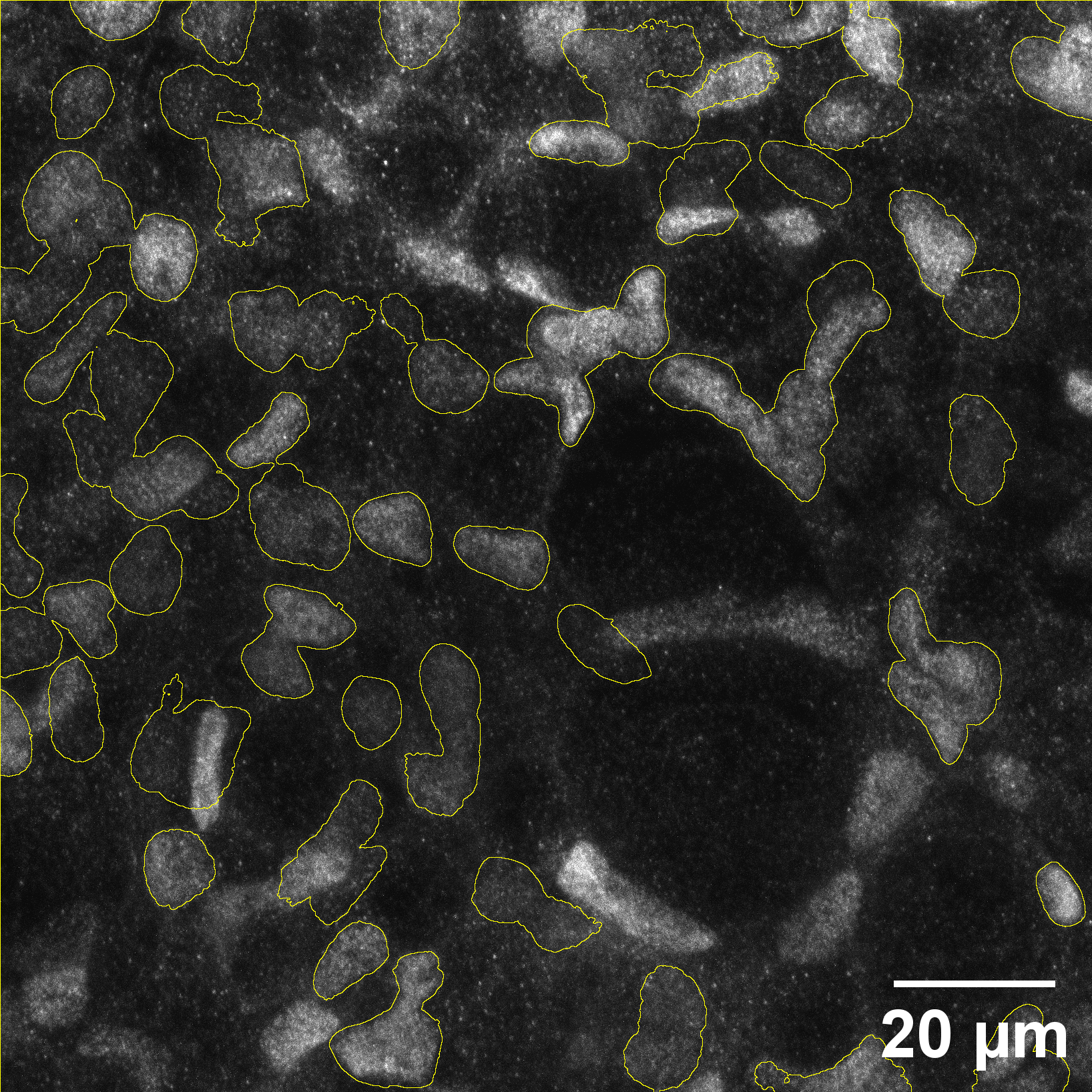

Supplement: Supplementary file 6 — Source data Fig. 5 [file 44319_2025_610_MOESM6_ESM.zip › Figure 5/5A/Fig5A_MIP_LN411_YAP.png]

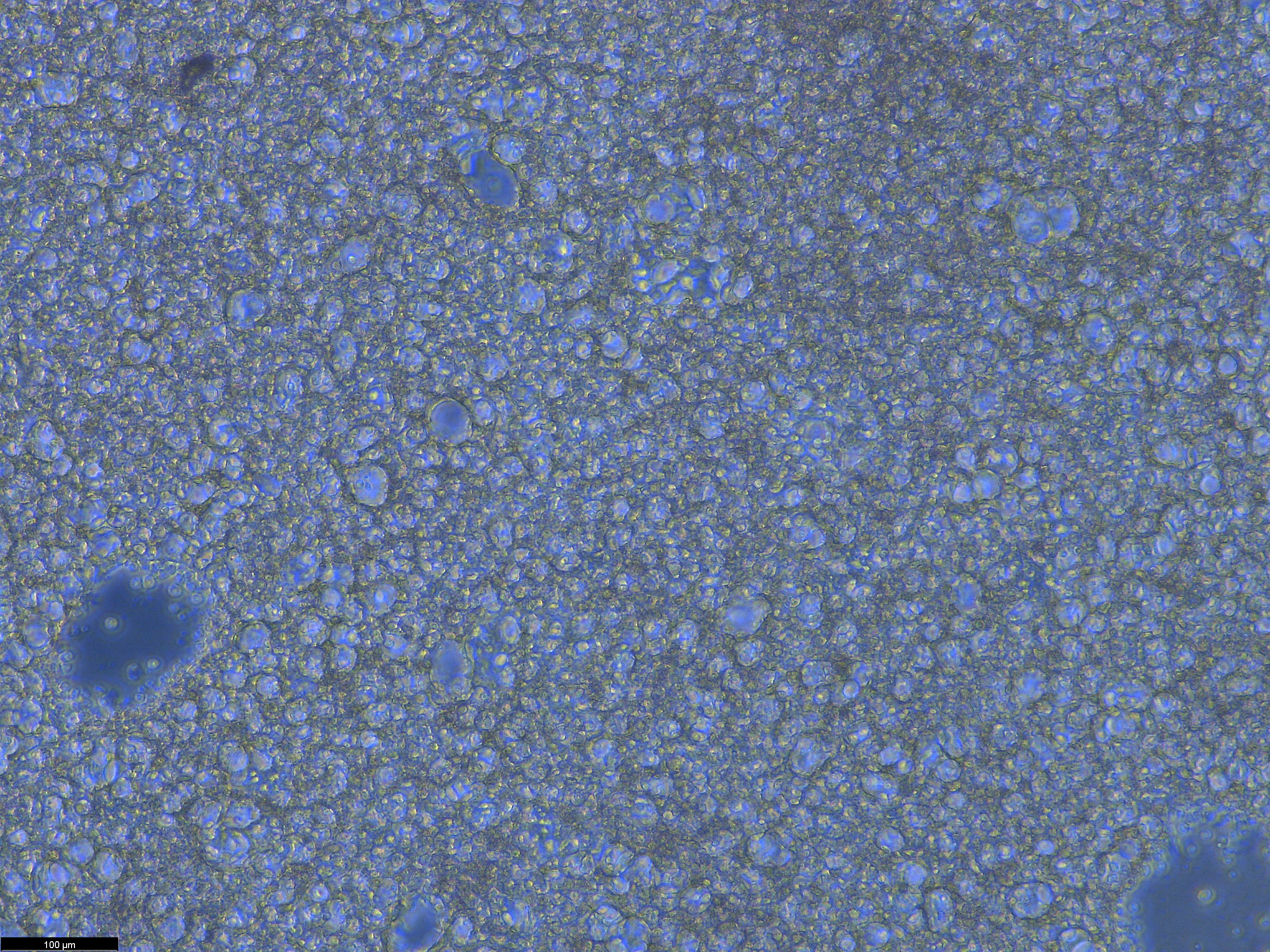

Supplement: Supplementary file 7 — Source data Fig. 6 [file 44319_2025_610_MOESM7_ESM.zip › Figure 6/6A/Fig6A_LN411Acetate.png]

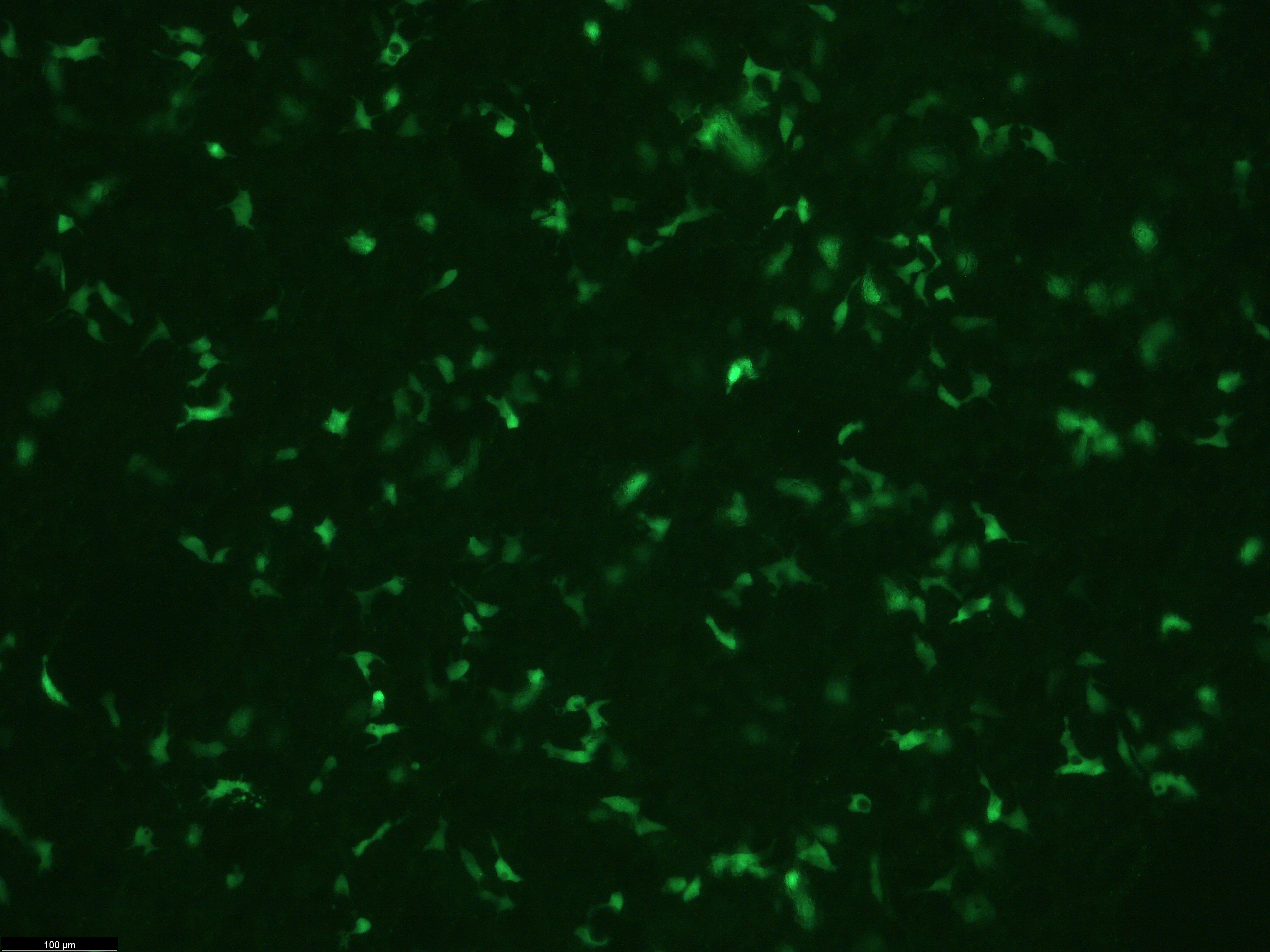

Supplement: Supplementary file 7 — Source data Fig. 6 [file 44319_2025_610_MOESM7_ESM.zip › Figure 6/6A/Fig6A_LN411AcetateGFP.png]

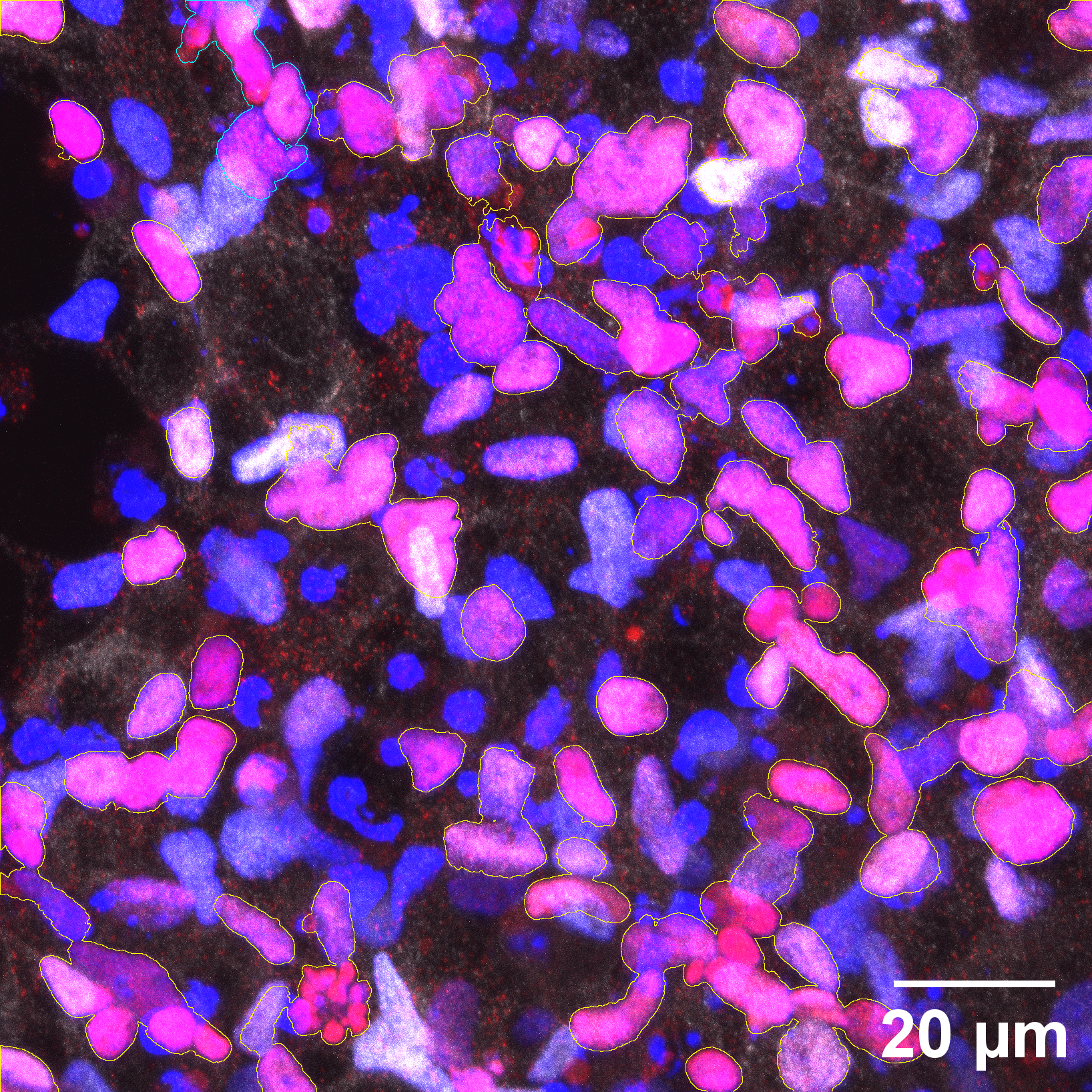

Supplement: Supplementary file 7 — Source data Fig. 6 [file 44319_2025_610_MOESM7_ESM.zip › Figure 6/6C/Fig6C_MIP_LN411Acetate_combined.png]

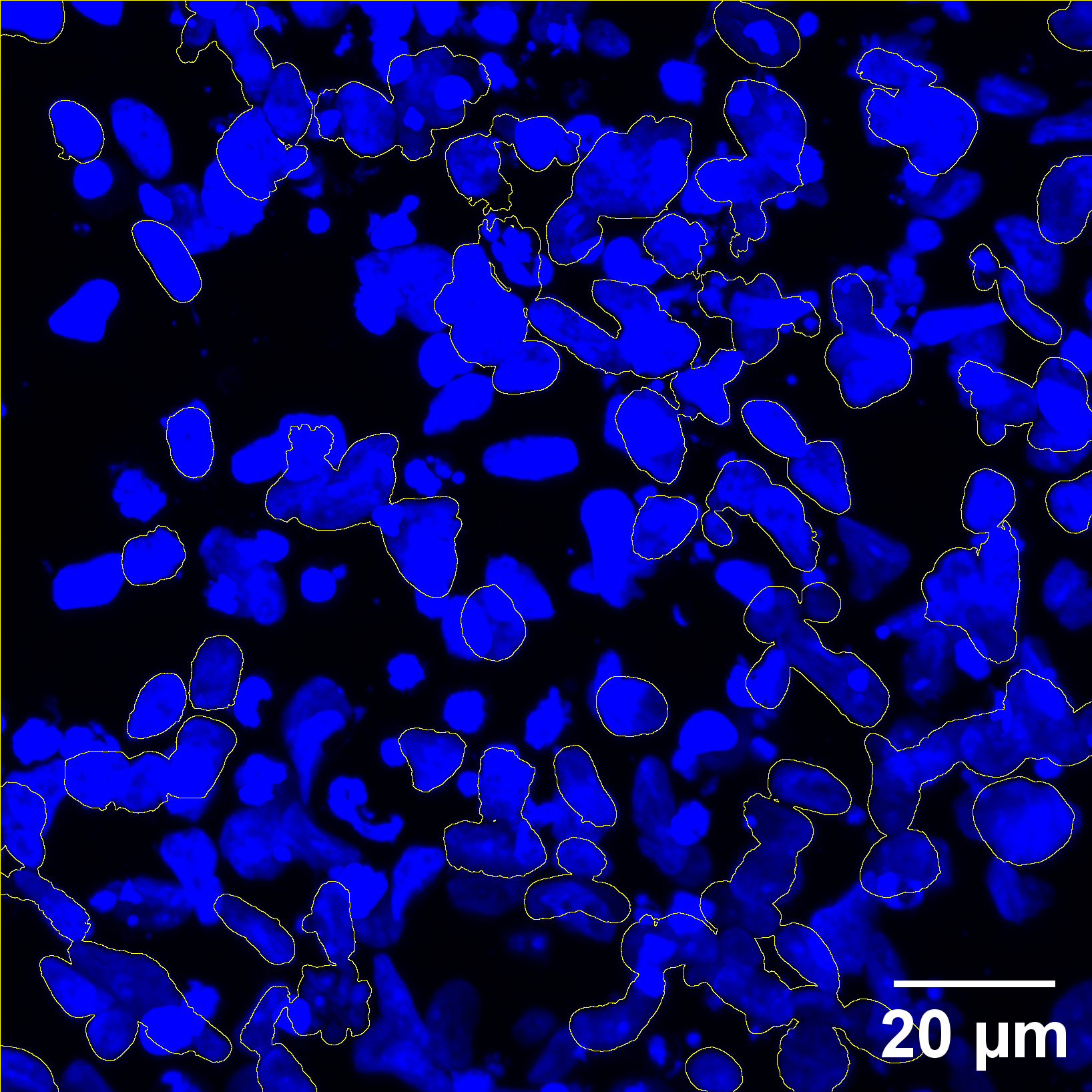

Supplement: Supplementary file 7 — Source data Fig. 6 [file 44319_2025_610_MOESM7_ESM.zip › Figure 6/6C/Fig6C_MIP_LN411Acetate_DAPI.png]

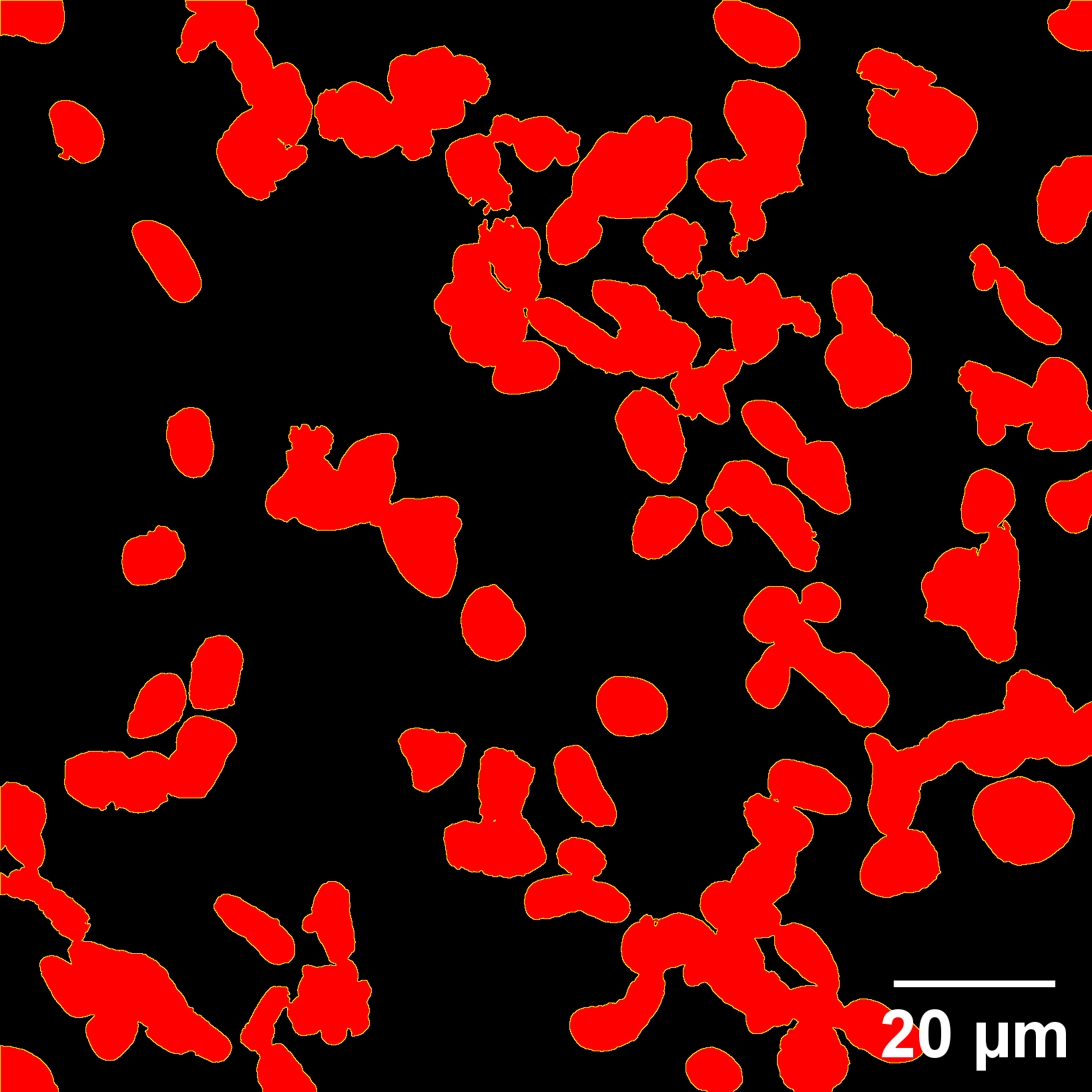

Supplement: Supplementary file 7 — Source data Fig. 6 [file 44319_2025_610_MOESM7_ESM.zip › Figure 6/6C/Fig6C_MIP_LN411Acetate_Mask.png]

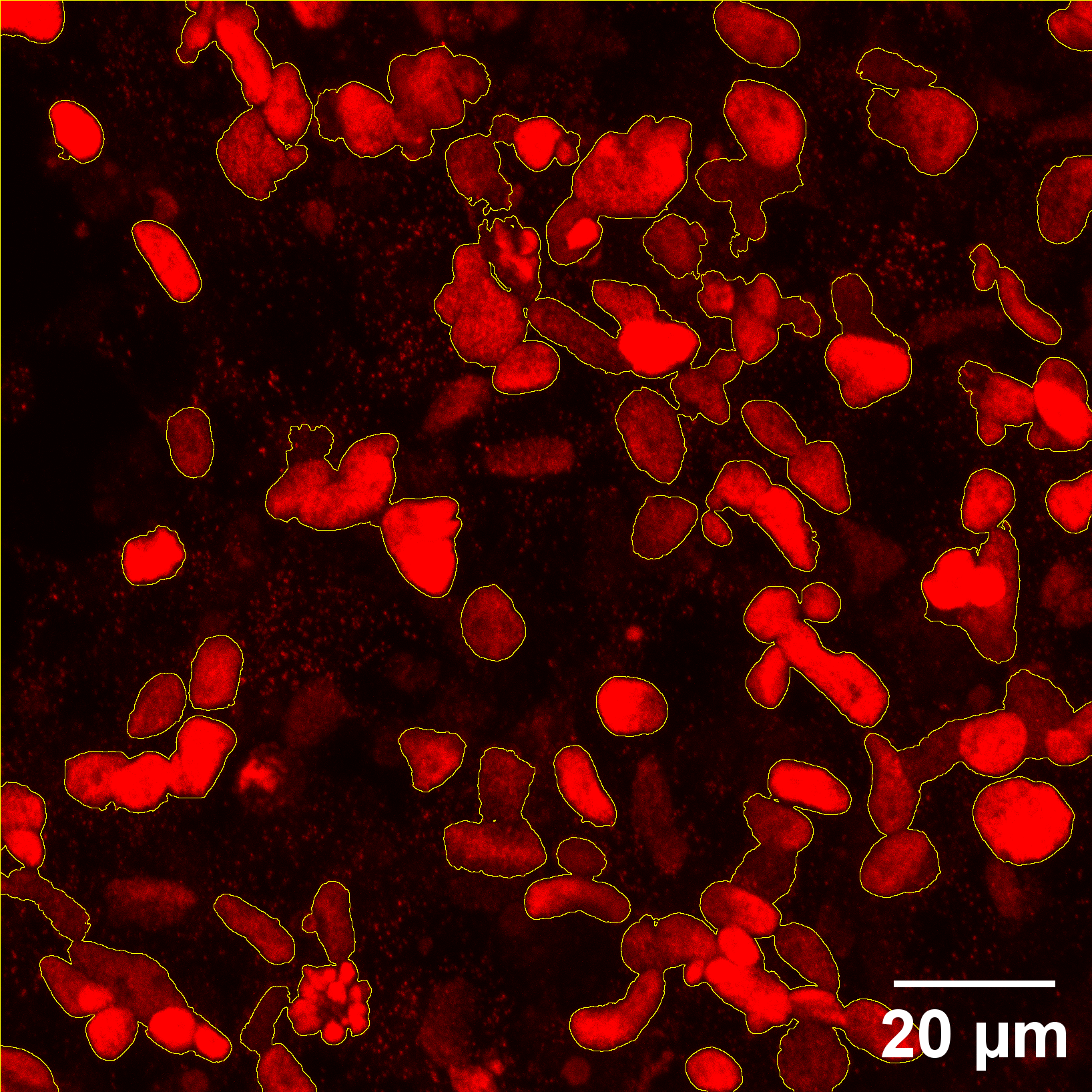

Supplement: Supplementary file 7 — Source data Fig. 6 [file 44319_2025_610_MOESM7_ESM.zip › Figure 6/6C/Fig6C_MIP_LN411Acetate_NKX61.png]

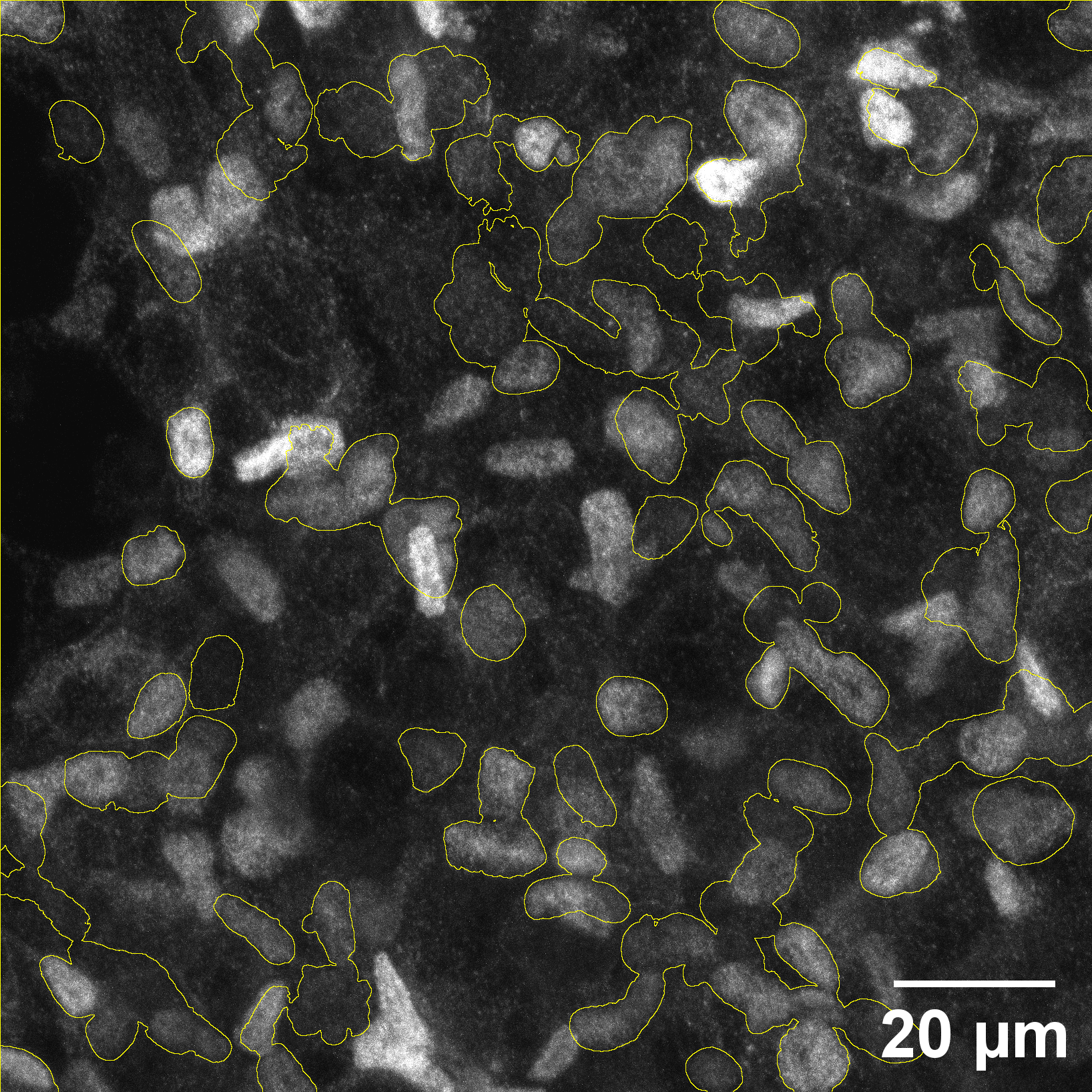

Supplement: Supplementary file 7 — Source data Fig. 6 [file 44319_2025_610_MOESM7_ESM.zip › Figure 6/6C/Fig6C_MIP_LN411Acetate_YAP.png]

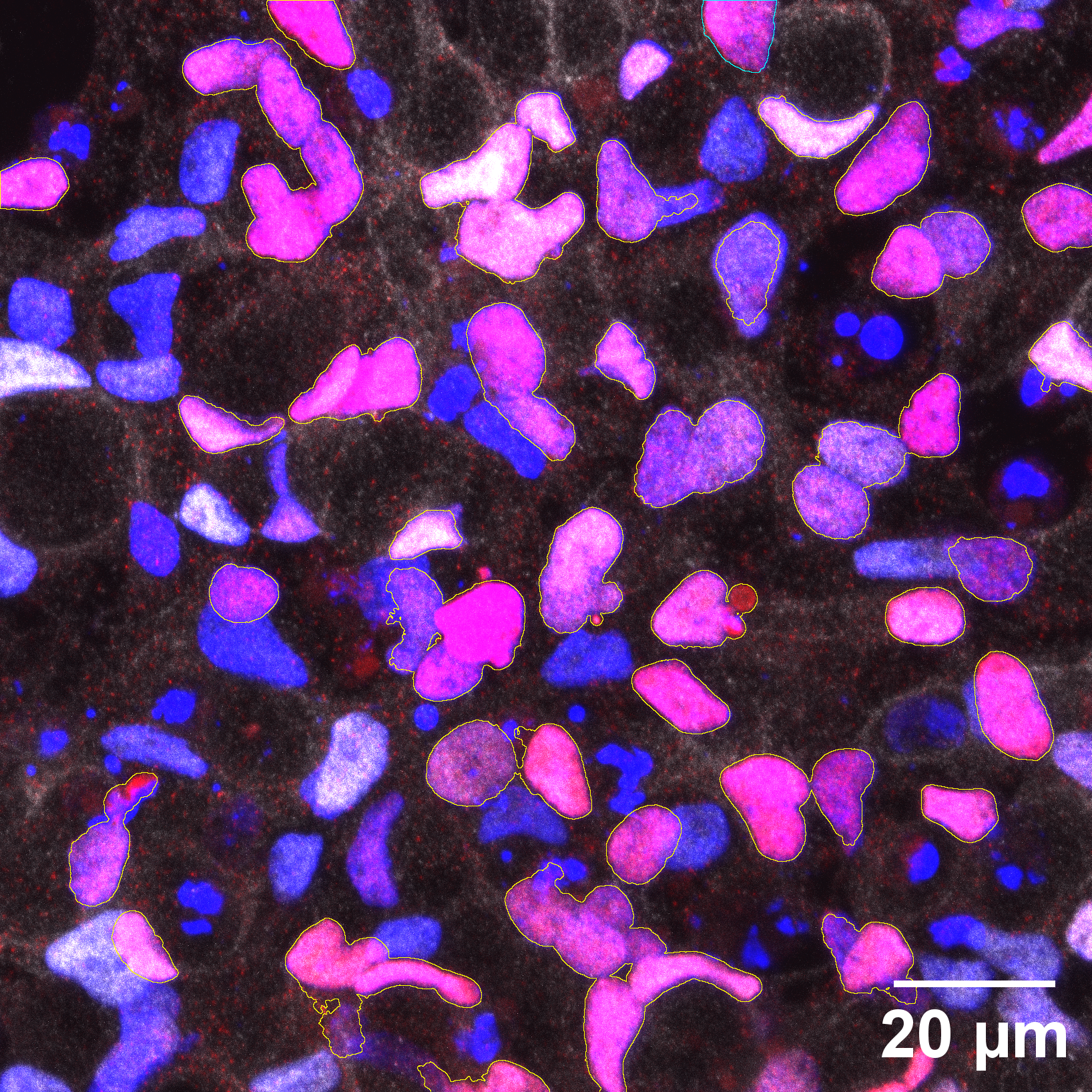

Supplement: Supplementary file 7 — Source data Fig. 6 [file 44319_2025_610_MOESM7_ESM.zip › Figure 6/6C/Fig6C_MIP_LN411_combined.png]

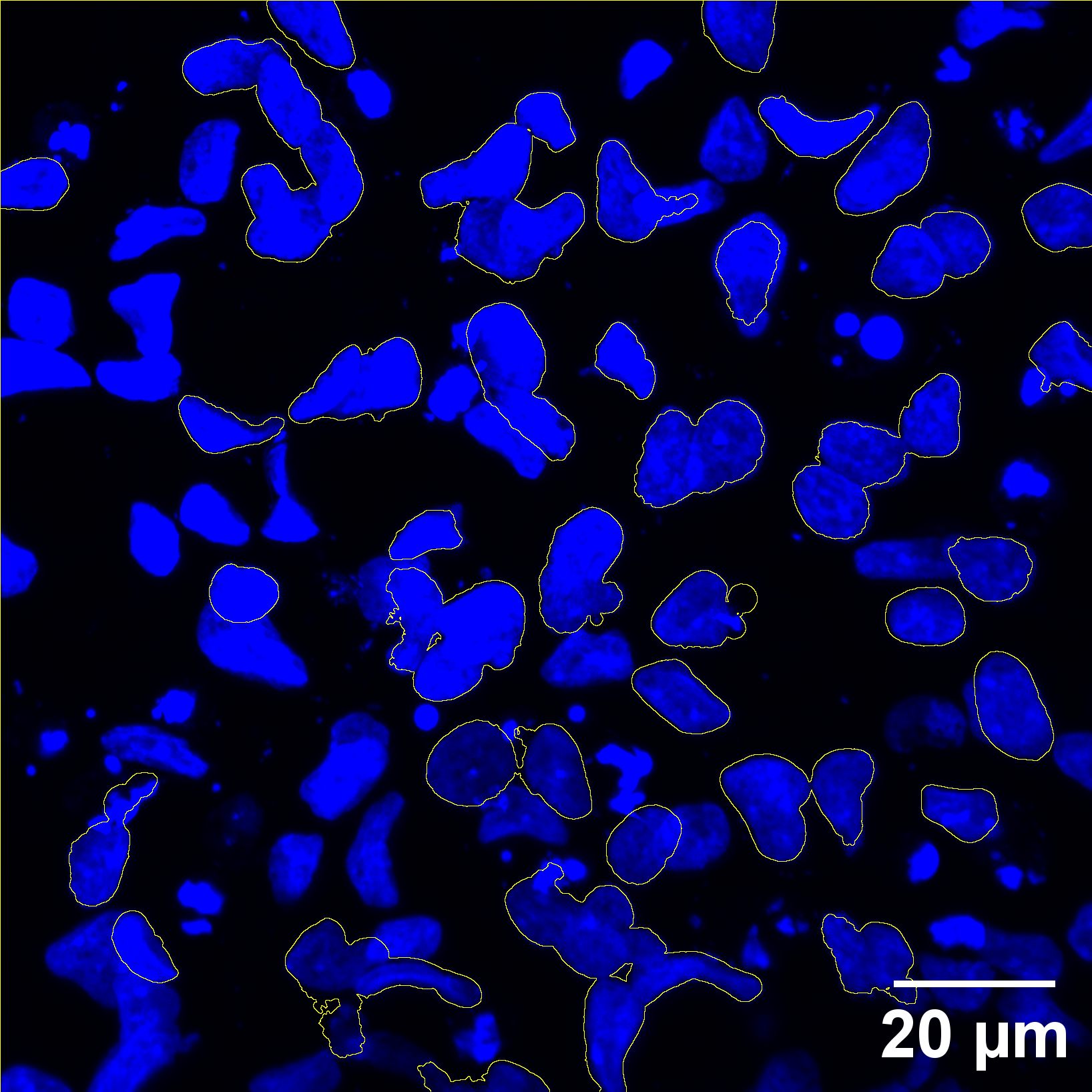

Supplement: Supplementary file 7 — Source data Fig. 6 [file 44319_2025_610_MOESM7_ESM.zip › Figure 6/6C/Fig6C_MIP_LN411_DAPI.png]

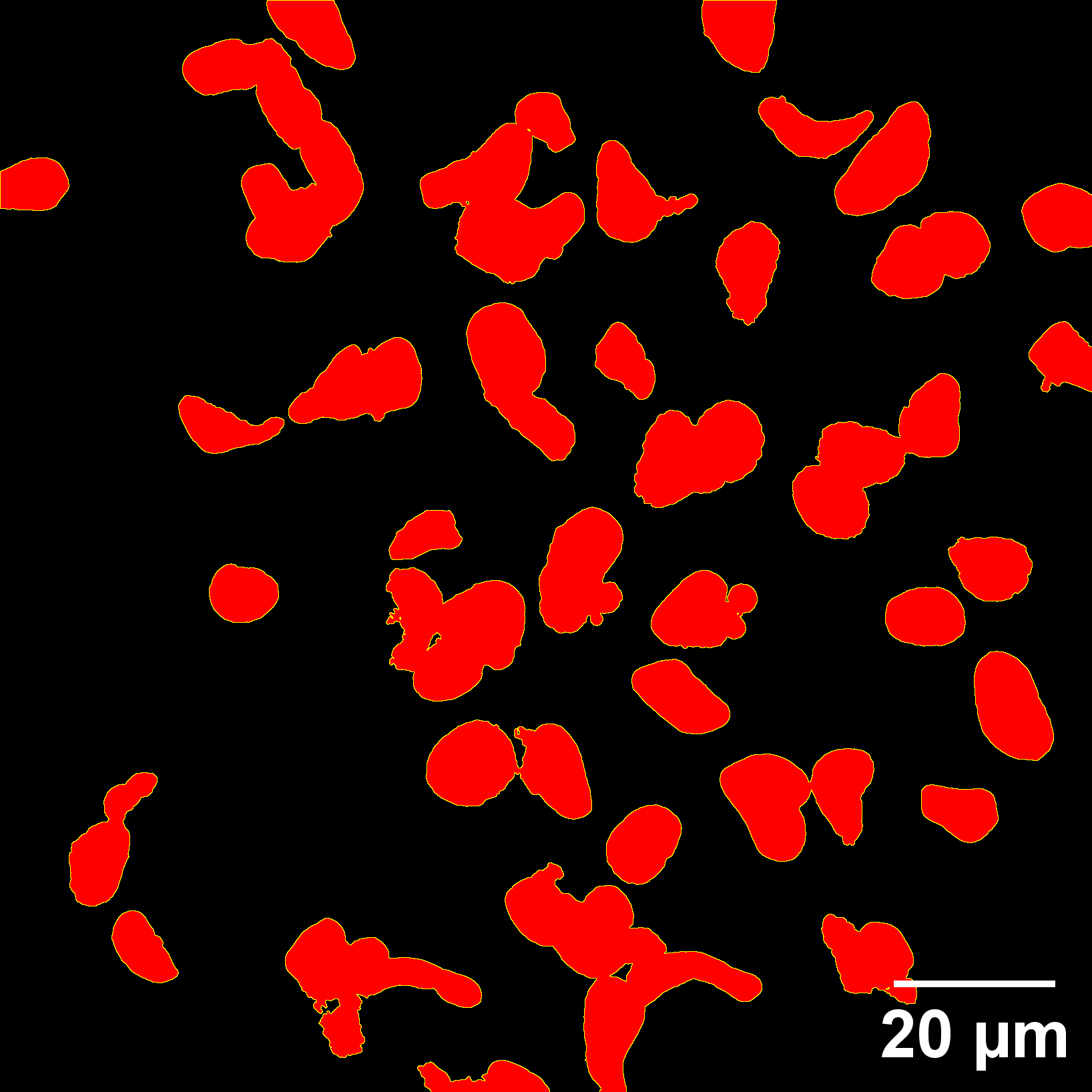

Supplement: Supplementary file 7 — Source data Fig. 6 [file 44319_2025_610_MOESM7_ESM.zip › Figure 6/6C/Fig6C_MIP_LN411_Mask.png]

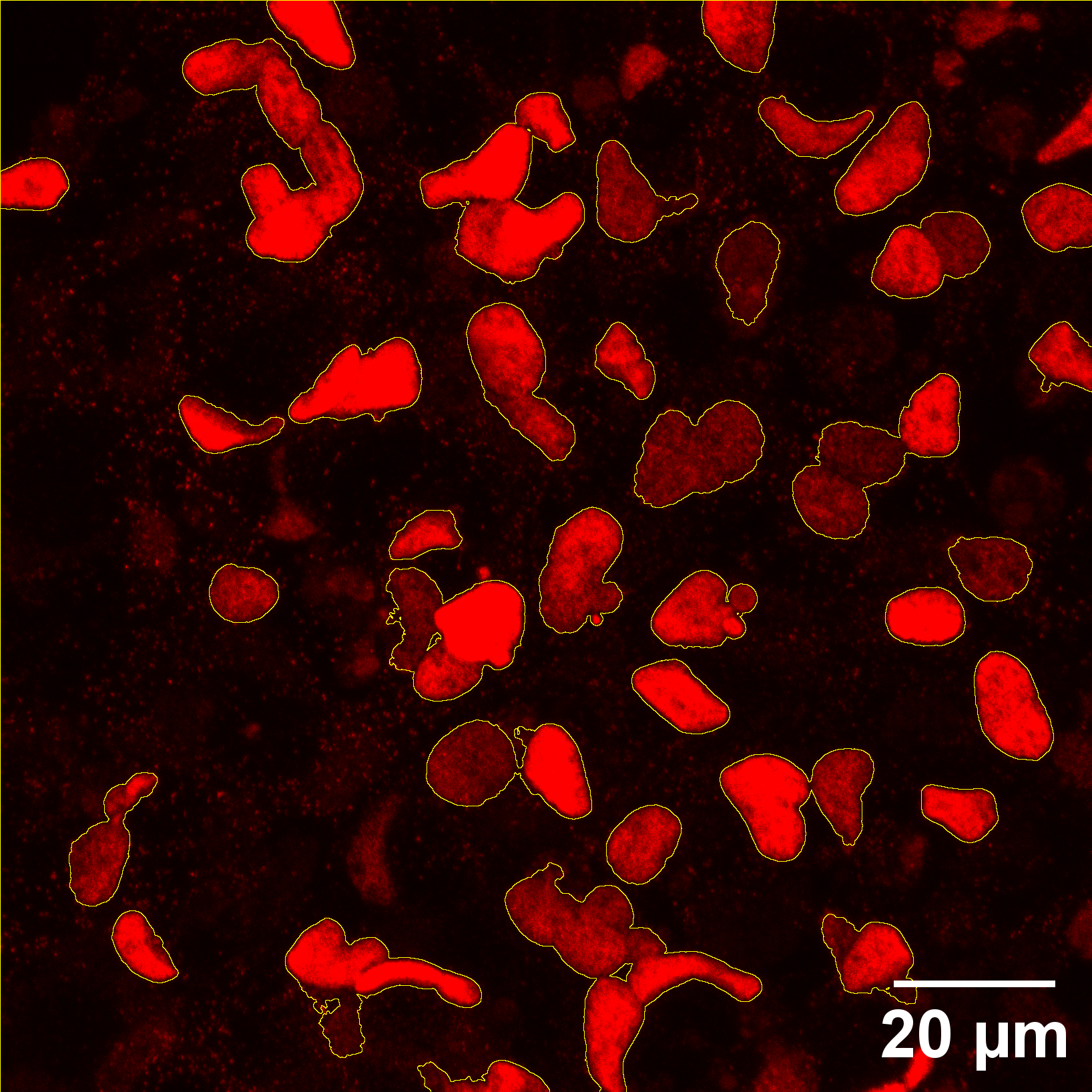

Supplement: Supplementary file 7 — Source data Fig. 6 [file 44319_2025_610_MOESM7_ESM.zip › Figure 6/6C/Fig6C_MIP_LN411_NKX61.png]

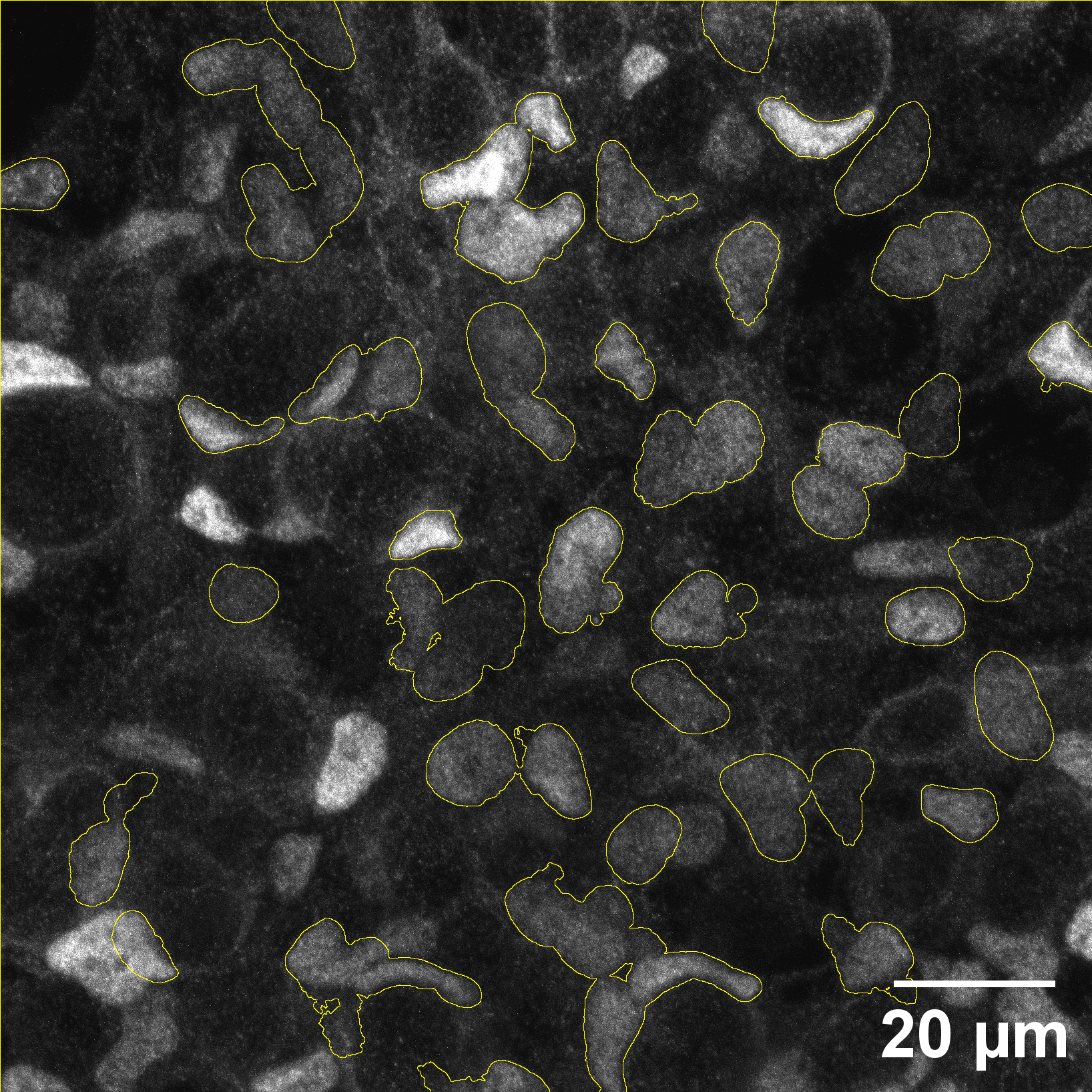

Supplement: Supplementary file 7 — Source data Fig. 6 [file 44319_2025_610_MOESM7_ESM.zip › Figure 6/6C/Fig6C_MIP_LN411_YAP.png]
