# Supplementary material for: Extracellular matrix-driven metabolic control of pancreatic endocrine lineage allocation
Source: EMBO Rep. 2025 Oct 27;26(23):5877–904. doi: 10.1038/s44319-025-00610-6 (PMC12678434; doi:10.1038/s44319-025-00610-6)
Supplement: Supplementary file 8 — Expanded View Figures [file 44319_2025_610_MOESM8_ESM.pdf]

Expanded View Figures

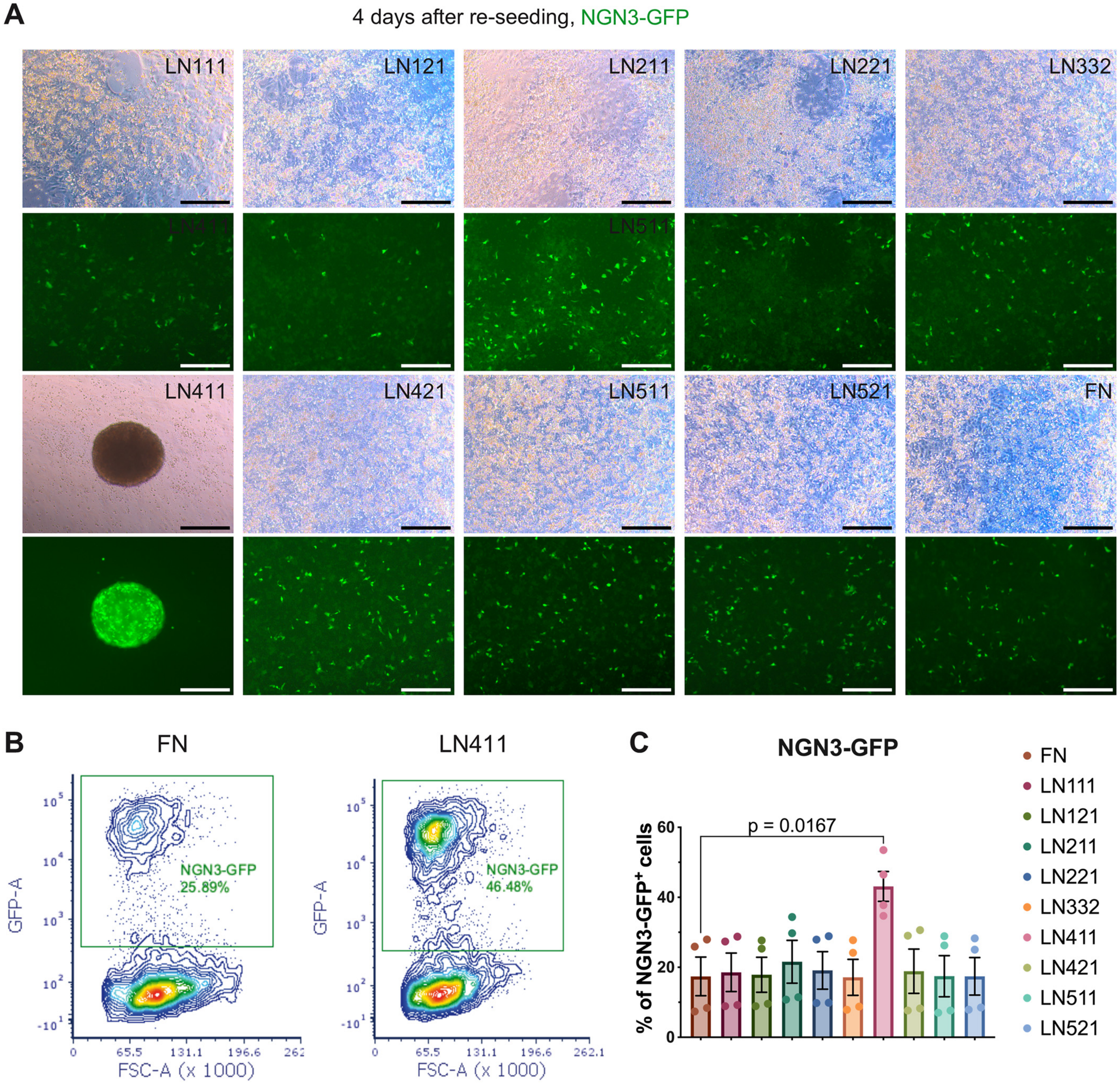

Figure EV1. LN411 specifically induces endocrine specification in the NGN3-GFP cell line.

(**A**) Representative brightfield (first and third row of panels) and epifluorescence (second and fourth row of panels) images of NGN3-GFP-derived pancreatic progenitors 4 days after reseeding on the indicated ECM proteins. Scale bar = 200 µm. (**B**) Representative flow cytometry plots of NGN3-GFP reporter expression 4 days after reseeding. (**C**) Quantification of NGN3-GFP reporter expression in (**B**). The data were analyzed by Dunnett's test with comparison to FN and are shown as mean expression ± SEM ($n = 4$, biological replicates).

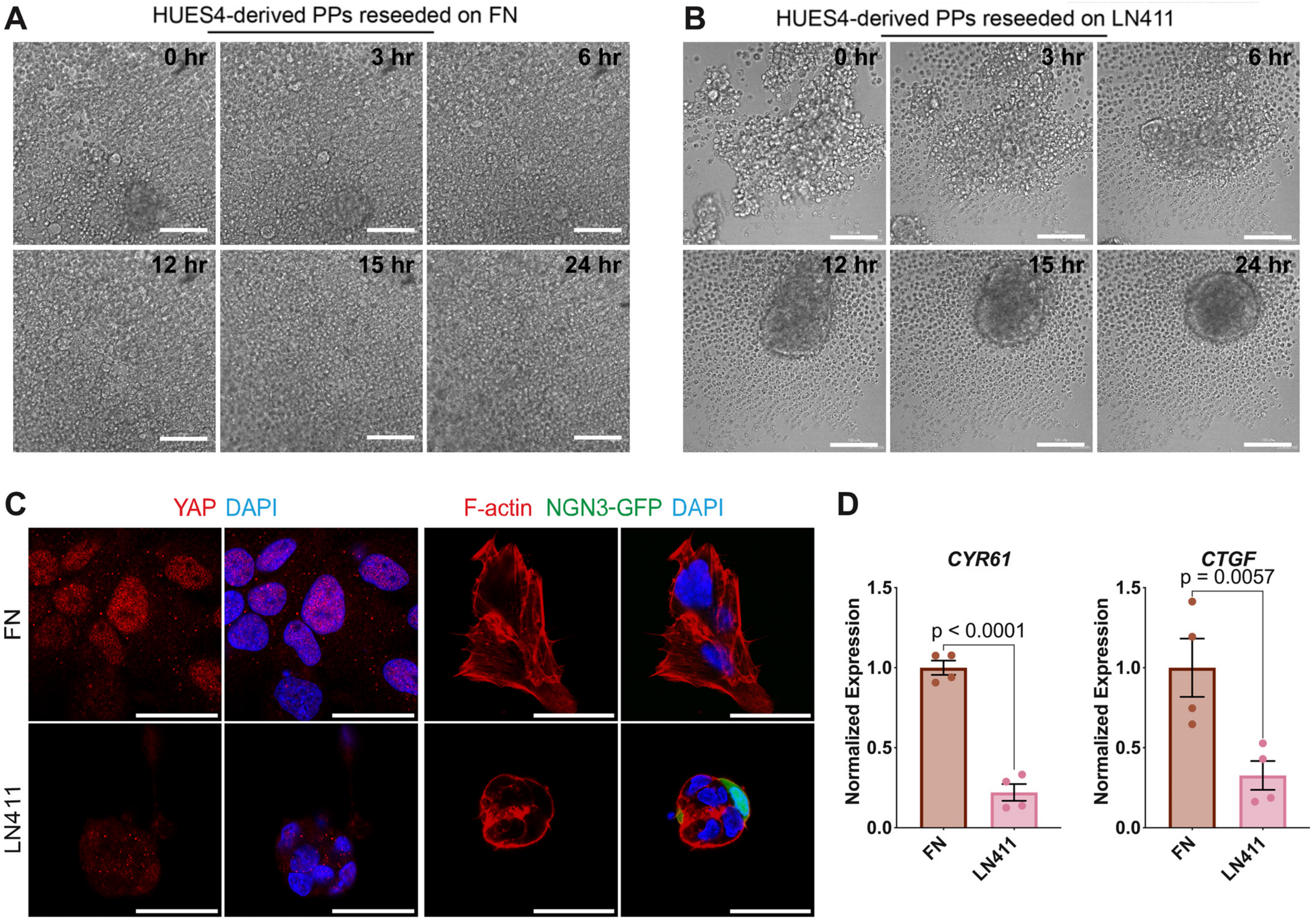

Figure EV2. LN411 induces a confined morphology, reduces actin polymerization, and decreases YAP activity.

(**A**) Live imaging of HUES4-derived pancreatic progenitors reseeded on FN by differential-interference contrast (DIC). Imaging started 1 h after reseeding. Scale bar = 100 μM. (**B**) Live imaging of HUES4-derived pancreatic progenitors reseeded on LN411 by differential-interference contrast (DIC). Imaging started 1 h after reseeding. Scale bar = 100 μM. (**C**) NGN3-GFP-derived pancreatic progenitors were sorted for GP2 using fluorescence-activated cell sorting, reseeded on FN (top panels) or LN411 (bottom panels) for 2 days and immunostained for YAP (left panels, red) or with phalloidin (right panels, red) in addition to DAPI (blue). Scale bar = 30 μm. (**D**) qPCR analysis of pancreatic of the YAP target genes *CYR61* and *CTGF* in NGN3-GFP derived pancreatic progenitors 24 h after reseeding on LN411 or FN. The data were analyzed using two-tailed paired t-tests and are shown as mean expression ± SEM ($n = 4$, biological replicates).

 © The Author(s)

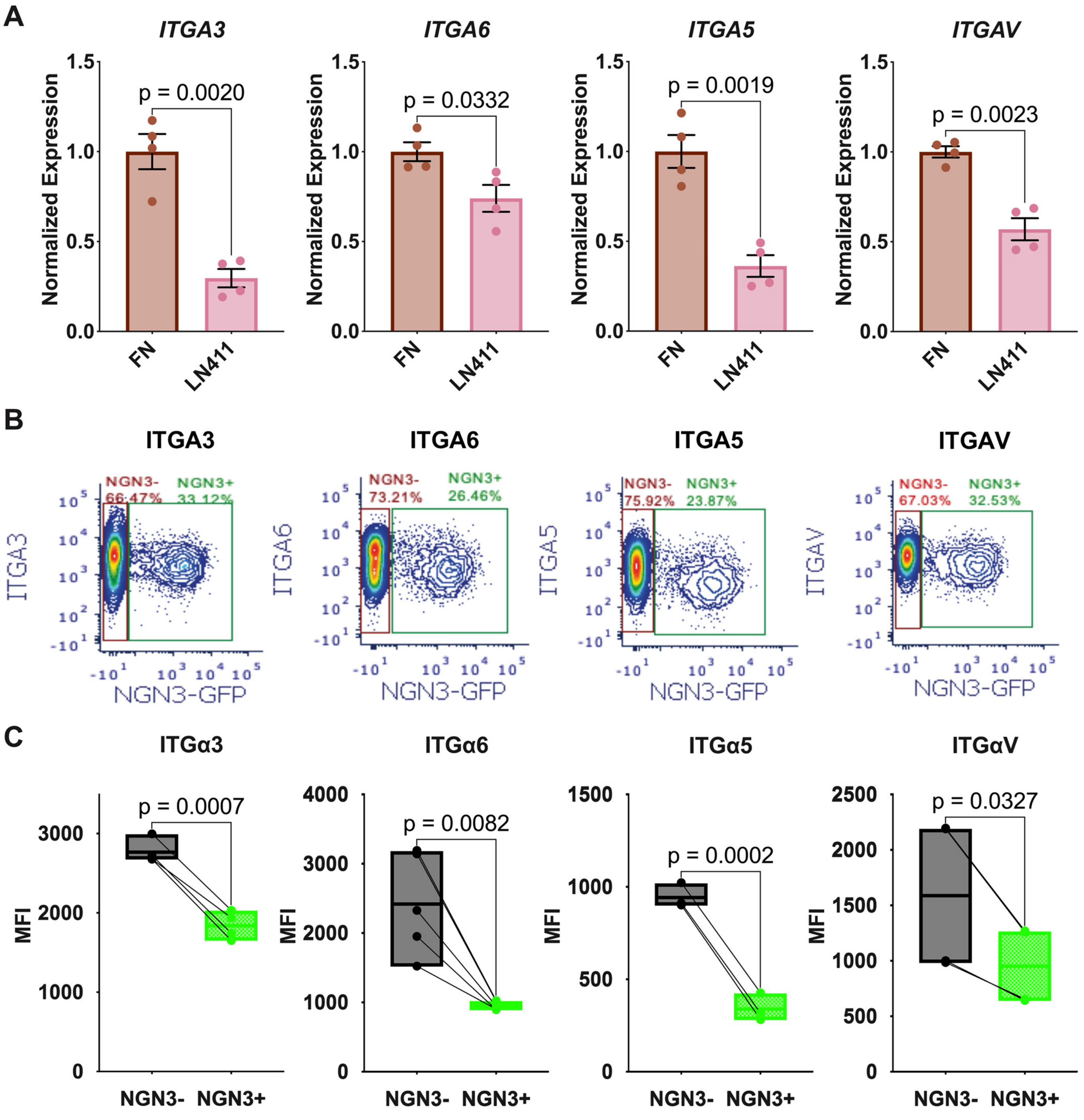

Figure EV3. Reseeding on LN411 leads to downregulation of integrins.

(**A**) qPCR analysis of indicated integrins in HUES4-derived pancreatic progenitors 4 days after reseeding on FN or LN411. The data were analyzed using two-tailed paired t-tests and are shown as mean expression ± SEM ($n = 4$, biological replicates). (**B**) Representative flow cytometry plots of Latrunculin B-treated NGN3-GFP-derived pancreatic progenitors immunostained for indicated integrins. (**C**) Quantification of mean fluorescence intensity in NGN3-GFP^{+} and NGN3-GFP^{-} cells as indicated in (**B**). Data were analyzed by two-tailed paired *t*- and are shown as boxplots with box boundaries extending to the minimum and maximum with a line at the mean ($n = 4$ for ITGα3 and ITGα6, $n = 3$ for ITGα5, $n = 2$ for ITGαV, biological replicates).

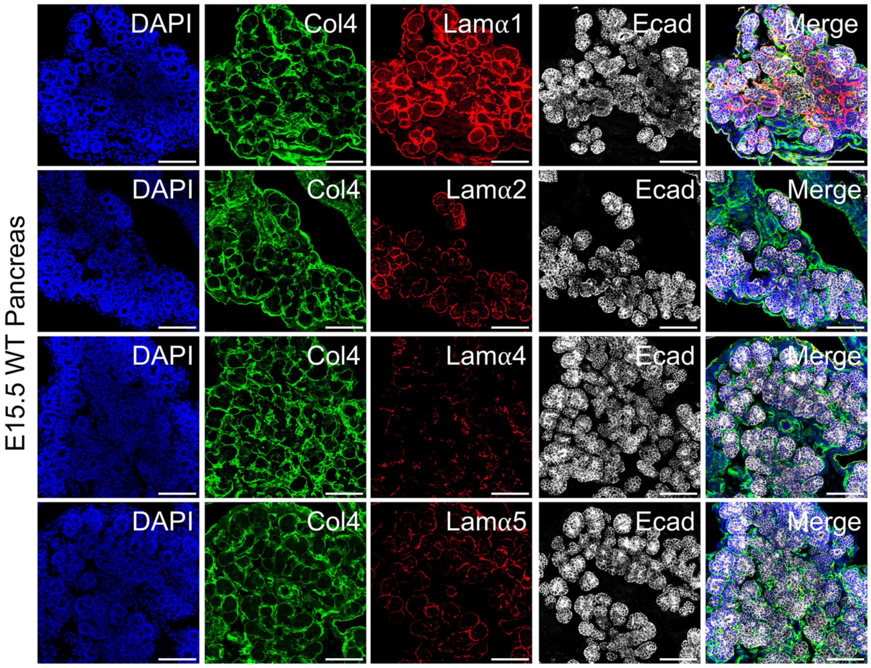

Figure EV4. Heterogenous expression of laminin isoforms in the pancreatic epithelium.

Immunofluorescent staining of 20-μm sections of E15.5 pancreas immunostained for collagen IV (Col4, green), E-cadherin (E-cad, white), and indicated laminin α chains (Lamα1-5, red), as well as DAPI (blue). Scale bar = 100 μM.

 © The Author(s)

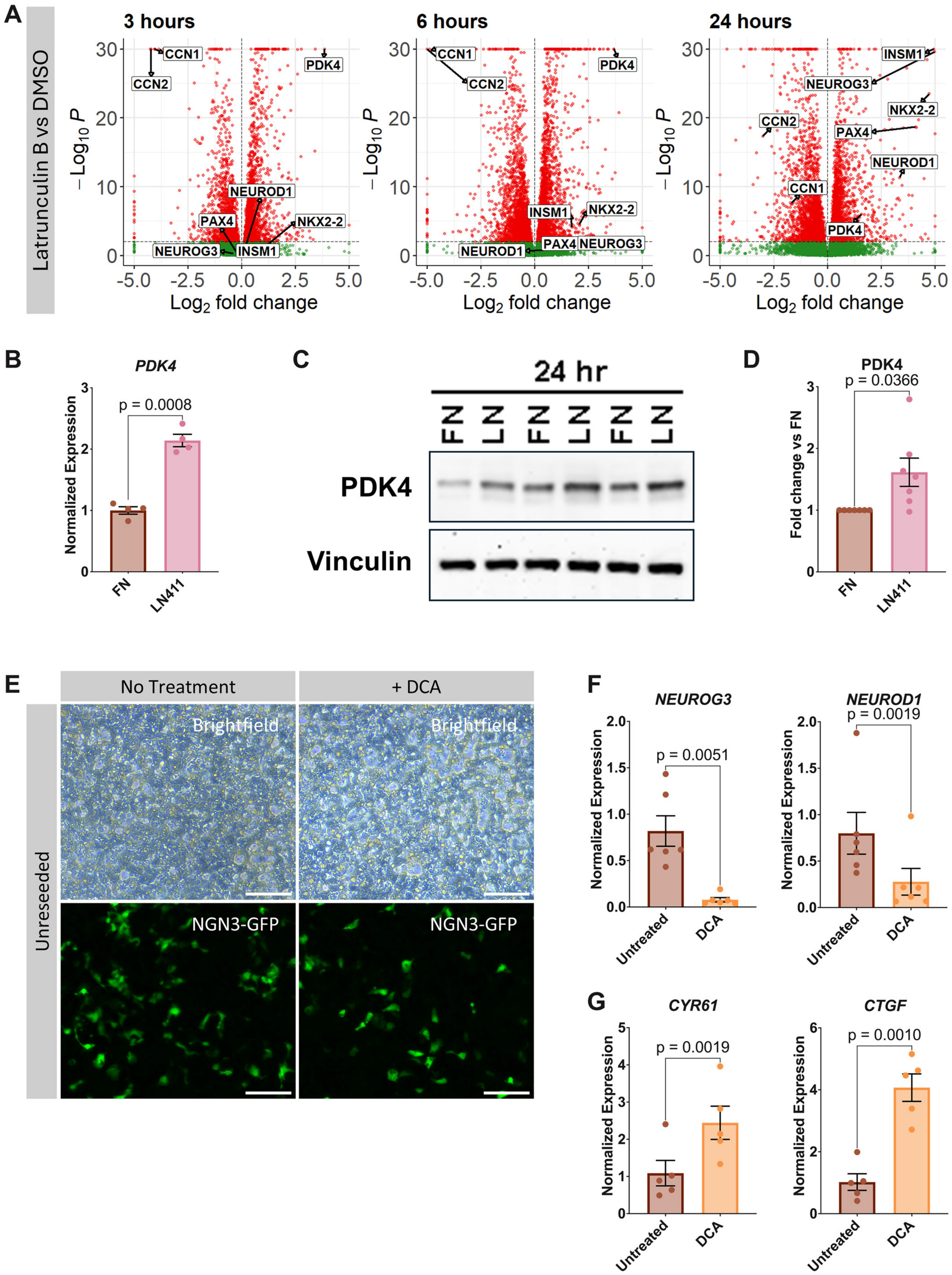
A
Latrunculin B vs DMSO
3 hours
6 hours
24 hours
CCN1
CCN2
PDK4
NEUROD1
PAX4
NKX2-2
NEUROG3
INSM1
−Log10 P
Log2 fold change
B
PDK4
p = 0.0008
Normalized Expression
FN
LN411
C
24 hr
FN LN FN LN FN LN
PDK4
Vinculin
D
PDK4
p = 0.0366
Fold change vs FN
FN
LN411
E
No Treatment
+ DCA
Unreseeded
Brightfield
NGN3-GFP
F
NEUROG3
p = 0.0051
NEUROD1
p = 0.0019
Normalized Expression
Untreated
DCA
G
CYR61
p = 0.0019
CTGF
p = 0.0010

Figure EV5. *PDK4* upregulation precedes endocrine specification in non-reseeded pancreatic progenitors.

(**A**) Volcano plots of bulk RNA-seq analysis of NGN3-GFP-derived pancreatic progenitors treated with latrunculin B or DMSO at indicated time points. Expression of YAP target genes (*CCN1/CYR61, CCN2/CTGF*), early endocrine genes (*NEUROG3, NEUROD1, INSM1, NKX2-2)* and *PDK4* are indicated. Differential expression analysis was performed using DESeq2 ($n = 3$). (**B**) qPCR analysis of *PDK4* expression 24 h after reseeding on FN or LN411. The data were analyzed using two-tailed paired *t*-tests and are shown as mean expression ± SEM ($n = 4$). (**C**) Western blot of PDK4 expression 24 h after reseeding on FN or LN411 (LN). Vinculin is included as a loading control. (**D**) Quantification of (**C**). The data were analyzed using two-tailed paired *t*-tests and are shown as mean expression ± SEM ($n = 7$). (**E**) Brightfield (top panels) and epifluorescence (bottom panels) images of pancreatic progenitors after 2 days of no treatment or treatment with 10 mM DCA. Scale bar = 100 µm. (**F**) qPCR analysis of endocrine marker genes *NEUROG3* and *NEUROD1* in pancreatic progenitors after 2 days of no treatment or treatment with 10 mM DCA. The data were analyzed using two-tailed paired *t*-tests and are shown as mean expression ± SEM ($n = 6$). (**G**) qPCR analysis of YAP target genes *CYR61* and *CTGF* in pancreatic progenitors after 2 days of no treatment or treatment with 10 mM DCA. The data were analyzed using two-tailed paired *t*-tests and are shown as mean expression ± SEM ($n = 6$).

 © The Author(s)

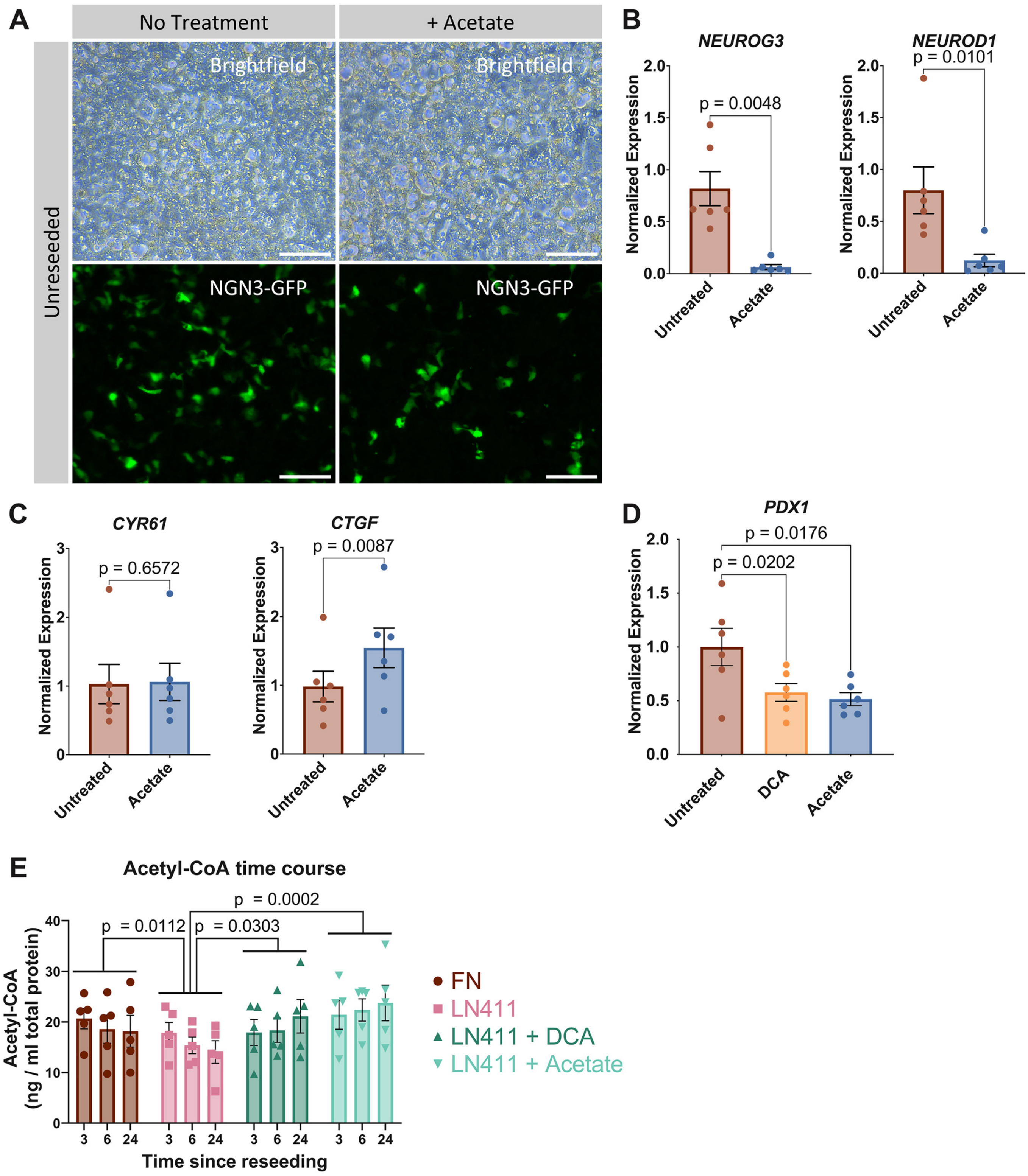
A
No Treatment
+ Acetate
Unreseeded
Brightfield
Brightfield
NGN3-GFP
NGN3-GFP
B
NEUROG3
p = 0.0048
NEUROD1
p = 0.0101
Normalized Expression
2.0
1.5
1.0
0.5
0.0
Untreated
Acetate
C
CYR61
p = 0.6572
CTGF
p = 0.0087
Normalized Expression
3
2
1
0
Untreated
Acetate
D
PDX1
p = 0.0176
p = 0.0202
Normalized Expression
Untreated
DCA
Acetate
E
Acetyl-CoA time course
p = 0.0002
p = 0.0112
p = 0.0303
Acetyl-CoA
(ng / ml total protein)
40
30
20
10
0
3 6 24
Time since reseeding
FN
LN411
LN411 + DCA
LN411 + Acetate

◀ **Figure EV6. Acetate inhibits endocrine specification via a YAP-independent mechanism.**

(**A**) Brightfield (top panels) and epifluorescence (bottom panels) images of NGN3-GFP-derived pancreatic progenitors after 2 days of no treatment or treatment with 20 mM sodium acetate. Scale bar = 100 µm. (**B**) qPCR analysis of endocrine marker genes *NEUROG3* and *NEUROD1* in pancreatic progenitors after 2 days of no treatment or treatment with 20 mM sodium acetate. The data were analyzed using two-tailed paired *t*-tests and are shown as mean expression ± SEM ($n = 6$). (**C**) qPCR analysis of YAP target genes *CYR61* and *CTGF* in pancreatic progenitors after 2 days of no treatment or treatment with 20 mM sodium acetate. The data were analyzed using two-tailed paired *t*-tests and are shown as mean expression ± SEM ($n = 6$). (**D**) qPCR analysis of *PDX1* in pancreatic progenitors after 2 days of no treatment or treatment with 10 mM DCA or 20 mM sodium acetate. The data were analyzed by Dunnett's test with comparison to the untreated condition and are shown as mean expression ± SEM ($n = 6$). (**E**) ELISA analysis of intracellular acetyl-CoA concentrations in pancreatic progenitor cells at 3, 6, and 24 h after reseeding on FN, LN411, or LN411 with 10 mM DCA or 20 mM sodium acetate. The data were analyzed by two-way repeated measures ANOVA testing for treatment differences across the entire time course (3–24 h), followed by Dunnett's test with comparison to LN411 alone and are shown as mean expression ± SEM ($n = 5$).

 © The Author(s)

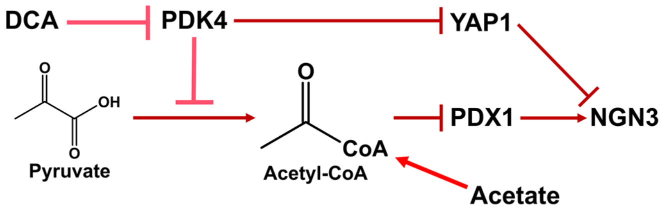

Figure EV7. PDK4 induces endocrine fate choice via both canonical and non-canonical functions.

Schematic illustrating the proposed method of PDK4-mediated endocrine fate choice. Canonically, PDK4 blocks conversion of pyruvate to acetyl-CoA via inhibition of PDH (not shown). PDK4 additionally blocks YAP activity through a non-canonical mechanism. PDX1 is required for *NGN3* expression, while YAP inhibits *NGN3* expression. DCA targets PDK4 directly and thus increases YAP activity and decreases *PDX1* expression. Acetate is converted to Acetyl-CoA, bypassing the effect of PDK4. Acetate treatment thus reduces *PDX1* expression without affecting YAP activity.

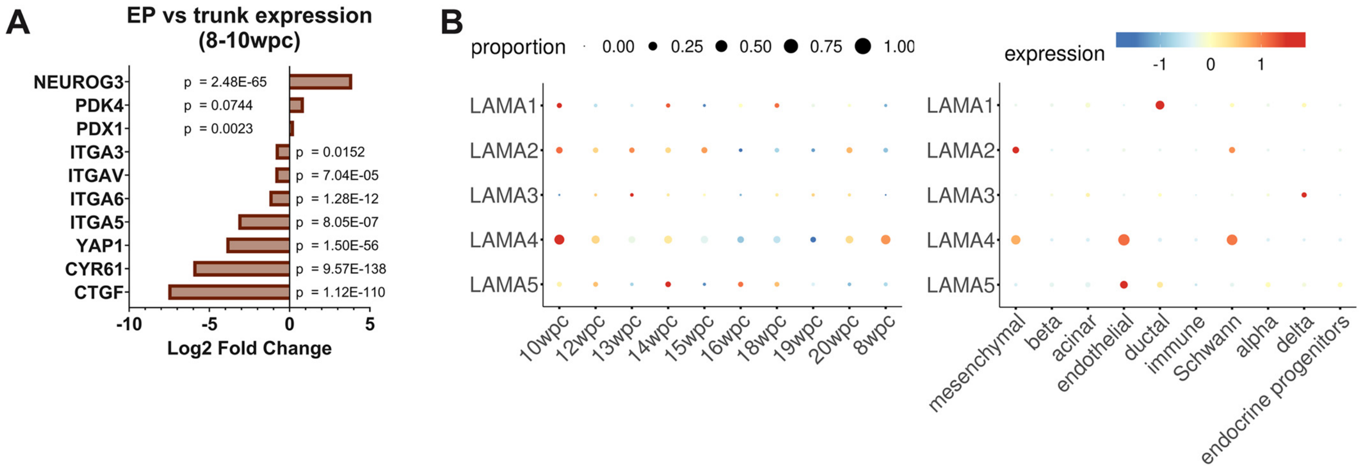

Figure EV8. Single-cell RNA sequencing datasets validate the LN411-PDK4-endocrine specification pathway.

(**A**) Log2 fold change in gene expression between endocrine progenitors (EP) and bipotent trunk progenitors (trunk) at 8–10 wpc in the OMIX001616 dataset (Ma et al, 2023). p-values are derived from MAST analysis (Finak et al, 2015) followed by Benjamini–Hochberg correction for multiple comparisons. (**B**) Dot plots of normalized expression of laminin α subunit genes in single-cell RNA sequencing samples of fetal pancreata according to developmental time (left) and cell type (right). Plots generated using humanpancreasdevelopment.org (Olaniru et al, 2023).

 © The Author(s)
